# Bioenergetic and inflammatory systemic phenotypes in Alzheimer’s disease APOE ε4‐carriers

**DOI:** 10.1111/acel.13356

**Published:** 2021-05-03

**Authors:** Heather M. Wilkins, Xiaowan Wang, Blaise W. Menta, Scott J. Koppel, Rebecca Bothwell, Annette M. Becker, Heidi Anderson, Erin Schwartz, Dong Pei, Nanda K. Yellapu, Prabhakar Chalise, Cynthia M. Gouvion, Mohammad Haeri, Jeffrey M. Burns, Russell H. Swerdlow

**Affiliations:** ^1^ Department of Neurology University of Kansas Medical Center Kansas City KS USA; ^2^ University of Kansas Alzheimer's Disease Center Kansas City KS USA; ^3^ Department of Biochemistry and Molecular Biology University of Kansas Medical Center Kansas City KS USA; ^4^ Department of Molecular and Integrative Physiology University of Kansas Medical Center Kansas City KS USA; ^5^ Department of Biostatistics and Data Science University of Kansas Medical Center Kansas City KS USA; ^6^ Department of Pathology & Laboratory Medicine University of Kansas Medical Center Kansas City KS USA

**Keywords:** Alzheimer's disease, APOE, bioenergetics, inflammation, mitochondria

## Abstract

We examined the impact of an *APOE ε4* genotype on Alzheimer's disease (AD) subject platelet and lymphocyte metabolism. Mean platelet mitochondrial cytochrome oxidase Vmax activity was lower in *APOE ε4* carriers and lymphocyte Annexin V, a marker of apoptosis, was significantly higher. Proteins that mediate mitophagy and energy sensing were higher in *APOE ε4* lymphocytes which could represent compensatory changes and recapitulate phenomena observed in post‐mortem AD brains. Analysis of the lipid synthesis pathway found higher AceCSI, ATP CL, and phosphorylated ACC levels in *APOE ε4* lymphocytes. Lymphocyte ACC changes were also observed in post‐mortem brain tissue. Lymphocyte RNAseq showed lower *APOE ε4* carrier sphingolipid Transporter 3 (SPNS3) and integrin Subunit Alpha 1 (ITGA1) expression. RNAseq pathway analysis revealed *APOE ε4* alleles activated inflammatory pathways and modulated bioenergetic signaling. These findings support a relationship between *APOE* genotype and bioenergetic pathways and indicate platelets and lymphocytes from *APOE ε4* carriers exist in a state of bioenergetic stress. Neither medication use nor brain‐localized AD histopathology can account for these findings, which define an *APOE ε4*‐determined molecular and systemic phenotype that informs AD etiology.

## Graphical abstract

APOE modulates Alzheimer's Disease Risk. We found that *APOE* genotype leads to a systemic phenotype. Lymphocytes and platelets from *APOE ε4* carriers show increased bioenergetic stress, mitochondrial dysfunction, apoptosis, and inflammation.

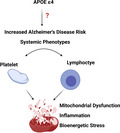

## 1 BACKGROUND

Apolipoprotein E (*APOE*) is the strongest genetic risk factor for sporadic Alzheimer's disease (AD). *APOE* exists as three alleles: ε2 (lowers AD risk), ε3 (neutral AD risk), and ε4 (increases AD risk). Individuals who carry an *APOE ε4* allele are threefold to fourfold more likely to develop AD, while homozygotes are 10‐ to 15‐fold more likely (Mahley & Huang, 2012). 15%–25% of the population carries an *APOE ε4* allele and 2%–3% are homozygous. The exact mechanism underlying the link between AD risk and *APOE* is unknown, although data suggest effects on mitochondria may mediate the association (Mahley & Huang, 2012).

The *APOE* gene product, APOE, is an apolipoprotein which binds lipids and functions in cholesterol metabolism. Within the brain, APOE is the main cholesterol‐carrying protein where it is mostly expressed by astrocytes and transports cholesterol to neurons (Mahley & Huang, 2012). Stress conditions cause neurons and microglia to express APOE, and conditions that activate APOE expression manifest in AD (Aoki et al., 2003). Allele variants lead to one or two amino acid substitutions in the protein product (APOE ε2, cys112, cys158; APOE ε3, cys112, arg158; and APOE ε4, arg112, arg158). The presence of arg112 in APOE ε4 causes an alternative folding of the peptide that gives rise to a cleavage site from which a mitochondrial‐toxic C‐terminal fragment is formed (Mahley & Huang, 2012). APOE also modulates inflammation and amyloid‐beta clearance (Dafnis et al., 2016; Mouchard et al., 2019; Wellnitz et al., 2005).

*APOE* genotype is associated with early changes (i.e., prior to dementia onset) in brain metabolism, cognition, and neuroimaging measures (Cavedo et al., 2017; Evans et al., 2014; Filippini et al., 2011; Mosconi et al., 2004). Systemic metabolic effects are also observed (Morris et al., 2017). We previously reported changes in blood‐based mitochondrial biomarkers in women *APOE ε4* carriers with AD. When compared to age‐matched non‐carrier women with AD, the AD *APOE ε4* carriers had decreased platelet cytochrome oxidase (COX) and citrate synthase (CS) enzyme maximum velocities (Vmax) (Wilkins et al., 2017). Reduced COX Vmax can cause bioenergetic stress including changes in redox balance and ATP production. An alternative encoding of COX subunits that perturbs holoenzyme structure/conformation, chemical inhibition, or lower mitochondrial mass could all contribute to this phenomenon.

Recent studies associate APOE genotype with mitochondria‐related endpoints. In post‐mortem human brain, *APOE ε4* carriers showed reduced synaptic proteins, reduced mitochondrial fusion/fission, biogenesis, and superoxide dismutase proteins irrespective of AD diagnosis (Yin et al., 2020). The presence or absence of an AD diagnosis is important to account for in studies such as this, because AD subjects typically use medications that non‐demented (ND) subjects do not. Critically, cholinesterase inhibitors that increase cholinergic signaling affect mitochondrial function (Kim et al., 2016), which could influence study findings and conclusions. Studies wanting to compare molecular signatures between groups need to consider medication effects. Here, we attempted to minimize the potential confounds of AD drugs by considering only *APOE ε4* carrier and non‐carrier AD subjects.

We now report an independent study of platelets and lymphocytes obtained from AD subjects. The platelet analysis was conducted as part of the S‐equol in AD 2 (SEAD2) clinical trial (ClinicalTrials.gov Identifier: NCT03101085), and the lymphocyte analysis is part of the “White Blood Cell Endpoints in AD” (WEAD) study we designed as an add‐on to SEAD2. Our goal was to characterize an *APOE ε4*‐defined, metabolism‐related molecular phenotype while avoiding the potential confounds of AD medications and AD brain histopathology.

## 2 MATERIALS AND METHODS

### 2.1 Approvals and human subjects

The Kansas University Medical Center Human Subjects Committee (KUMC HSC) approved all human subject participation, and all participants provided informed consent prior to enrolling. This study was conducted in accordance with the Code of Ethics of the World Medical Association (the Declaration of Helsinki). We enrolled participants who met McKhann et al. AD diagnostic criteria (McKhann et al., 2011). Participants were excluded if they reported any potentially confounding, serious medical risks such as type 1 diabetes, cancer, or a recent cardiac event such as a heart attack or angioplasty. At the beginning of the study, the trial participants underwent a 40 ml phlebotomy.

Autopsy brain samples were obtained from the KU Alzheimer's Disease Research Center (KU ADRC) Neuropathology Core. The KU ADRC maintains a clinical cohort and collects brains from consenting cohort decedents. The autopsy consent process is approved by the KUMC HSC.

### 2.2 Phlebotomy and blood cell separation

Forty millilitre of blood was collected in tubes containing acid‐citrate‐dextrose anticoagulant. One millilitre of whole blood was removed and stored at −80°C for genotyping; the rest was used for platelet and lymphocyte harvesting.

Fifteen millilitre of Histopaque 1077 was centrifuged in an AccuSpin tube for 1 min at 1700 *g*. Blood was layered on top of the AccuSpin tube frit and centrifuged for 15 min at 400 *g*. Platelet‐rich plasma and the buffy coat were collected in separate tubes and pelleted by centrifugation for 15 min at 1700 *g*. The pellets were washed with phosphate‐buffered saline (PBS) and re‐centrifuged. Platelets were used for mitochondrial isolation. Lymphocytes were used for flow cytometry biomarkers and tissue culture expansion. Blood samples were processed on the same day as the blood draw.

### 2.3 APOE genotyping

We used a single nucleotide polymorphism (SNP) allelic discrimination assay to determine *APOE* genotypes. This involved adding 5 µl of blood to a TaqMan Sample‐to‐SNP kit (Thermo Fisher). TaqMan probes to the two *APOE*‐defining SNPs, rs429358 (C_3084793_20) and rs7412 (C_904973_10) (Thermo Fisher), were used to identify *APOE ε2*, *ε3*, and *ε4* alleles.

### 2.4 Mitochondrial isolation

Platelets were resuspended in MSHE buffer (225 mM mannitol, 75 mM sucrose, 5 mM HEPES, 1 mM EGTA, pH 7.4) and disrupted by nitrogen cavitation, at 1200 psi, for 20 min. The ruptured platelets were centrifuged at 1000 *g* for 15 min, 4°C. The supernatant was transferred to a new tube, while the pellet (intact platelets) was resuspended in MSHE buffer and subjected to nitrogen cavitation for a second time (1200 psi for 20 min). Both supernatants were combined and centrifuged at 12,000 *g* for 10 min, 4°C. The resulting mitochondrial pellet was resuspended in MSHE buffer.

### 2.5 COX and CS Vmax assays

We added aliquots of the enriched platelet mitochondrial suspensions to cuvettes and spectrophotometrically determined each suspension's COX and CS Vmax activities. For the COX Vmax, we followed the conversion of reduced cytochrome *c* to oxidized cytochrome *c* and calculated the pseudo‐first‐order rate constant (m/s). For the CS Vmax, we followed the formation of 5‐thio‐2‐nitrobenzoate (nmol/min). The COX rate was normalized to mg protein (m/s/mg protein) or to the CS rate (yielding a value with units of m/s/nmol/min), which we herein refer to simply as COX/CS. The CS rate was normalized to mg protein to yield a final activity with units of nmol/min/mg protein.

### 2.6 Dye‐based assays and flow cytometry

Fresh lymphocytes were suspended in HBSS (with Ca^2+^/Mg^2+^) at 1 × 10^6^/ml. Four millilitre of lymphocytes were used for negative (no stain), JC1, MitoSox, and MitoTracker/Annexin V staining. For staining, we used 10 μl of 200 μM JC1, 4 μl of 10 μM MitoTracker Red, 2 μl of 5 mM MitoSox, or no dye. Cells were incubated at 37°C with 5% CO_2_ for 30 min. All samples were centrifuged to pellet cells (1700 *g* for 5 min). Cells were washed with HBSS and centrifuged again (1700 *g* for 5 min). The supernatants were removed, and 500 μL of HBSS was added to each tube (for all samples except the MitoTracker/Annexin V samples). For MitoTracker/Annexin V samples, 100 μL of 1X Annexin V binding buffer was added with 5 μL of Annexin V dye. MitoTracker/Annexin V samples were incubated at room temperature for 15 min, following which 400 μL of 1X Annexin V binding buffer was added. All samples were placed on ice and immediately analyzed using an LSRII flow cytometer (BD Bioscience).

### 2.7 Lymphocyte culture

Lymphocytes were resuspended in complete RPMI medium (RPMI, 10% FBS, Pen/Strep, 20 U/ml IL‐2, and 20 ng/ml CD3) at 1 × 10^6^ cells/ml in a T75 culture flask. Cells were fed every other day and split as needed to keep cell concentrations at 1 × 10^6^ cells/ml. Cells were used for immunochemistry as described below after 7 days of culture.

### 2.8 Immunochemistry

For expanded lymphocyte cultures and autopsied human brain superior frontal gyrus tissue sections, protein was collected in RIPA buffer with protease/phosphatase inhibitors (Thermo Fisher). Equal protein amounts were resolved by SDS‐PAGE (Criterion TGX gels, Bio‐Rad), and proteins were transferred to PVDF membranes (Thermo Fisher). Immunoblots were completed with antibodies listed in Table S1. To visualize bands, we used WestFemto Super Signal HRP Substrate (Thermo Fisher) and a ChemiDoc XRS imaging platform.

### 2.9 RNAseq and qPCR

Expanded lymphocyte cultures were dissolved in 1 ml of TRI reagent and incubated for ten minutes on ice. RNA was isolated via phenol‐chloroform extraction with TRI reagent. RNA purity and content were measured by A260/280 ratio.

Stranded Total RNA‐Seq was performed using an Illumina NovaSeq 6000 Sequencing System. Total RNA (input range: 1000 ng) was used to initiate the Stranded Total RNA‐Seq library preparation protocol. The total RNA fraction underwent ribosomal reduction, size fragmentation (6, 4, 3, or 2 min based on %DV_200_ calculation), reverse transcription into cDNA, and ligation with the appropriate‐indexed adaptors using the TruSeq Stranded Total RNA HT Sample Preparation Kit (Illumina #RS‐122‐2203). Following Agilent Bioanalyzer QC of the library preparation and library quantification using the Roche Lightcycler96 with FastStart Essential DNA Green Master Mix (Roche #06402712001), the RNAseq libraries were adjusted to a 2 nM concentration and pooled for multiplexed sequencing on a NovaSeq 6000. The onboard clonal clustering procedure was automated during the NovaSeq 6000 sequencing run. The 100‐cycle paired‐end sequencing was performed using the NovaSeq 6000 S1 Reagent Kit ‐ 200 cycle (Illumina #20012864). Following collection, sequence data were converted from.bcl file format to fastQ files and de‐multiplexed into individual sequences for further downstream analyses.

### 2.10 RNAseq data quality assessment and analysis

RNA‐Seq data quality was assessed through the FastQC tool (http://www.bioinformatics.babraham.ac.uk/projects/fastqc/). Paired‐end reads were mapped to the human genome (UCSC‐hg38 version) using RNAseq by Expectation‐Maximization (RSEM) (Li & Dewey, 2011). The Bowtie2 (Langmead & Salzberg, 2012) tool was used to align the reads to the reference genome, and the expression was calculated for the aligned reads. The expression quantity profiles were then used to generate an expression matrix of the samples.

Genes with no or low transcription, defined as <1 count per million (CPM) in 2 or more of 20 samples, were filtered out. After filtering the non/low expressed genes, a total of 15,677 genes were retained for downstream statistical analyses. Raw gene counts were then normalized according to library size using the TMM method as implemented in Bioconductor R package “edgeR” (Robinson et al., 2010). The differences in gene expression between the *APOE ε4* carriers and non‐carriers were assessed using the exact test based on negative binomial distribution using R package “edgeR”. The multiple testing adjustments were carried out using Benjamini and Hochberg's false discovery rate (FDR) method.

### 2.11 Pathways analysis

KEGG pathway analysis and visualization were performed using the R Bioconductor packages “gage” and “pathview” (Luo & Brouwer, 2013; Luo et al., 2009) using the results obtained from the differential expression (DE) analysis for the genes. To identify biological pathways enriched with differentially expressed genes, we performed a gene set enrichment analysis (GSEA) (Subramanian et al., 2005). Datasets were analyzed in reference to the msigdb.v7.2.symbols.gmt gene set database of the Molecular Signature Database (MSigDB) (Liberzon et al., 2011). Network activation analysis among the top 2,000 differentially expressed genes was also performed by Ingenuity Pathway Analysis (Qiagen Inc., https://www.qiagenbioinformatics.com/products/ingenuity‐pathway‐analysis). Analysis was performed in reference to the Ingenuity Knowledge Base including direct and indirect interactions with filters applied for human and blood cells. In addition, we also carried out Gene Ontology (GO) analyses using the DE results.

### 2.12 Clinical data and statistical analyses

We organized the data from the participants into two groups, one in which participants had at least one *APOE ε4* allele, and one in which they did not. Differences in means for each continuous outcome between the two groups were assessed using two‐sample *t* tests or chi‐squared test. A *p* value of <0.05 was considered statistically significant.

## 3 RESULTS

**FIGURE 1:**
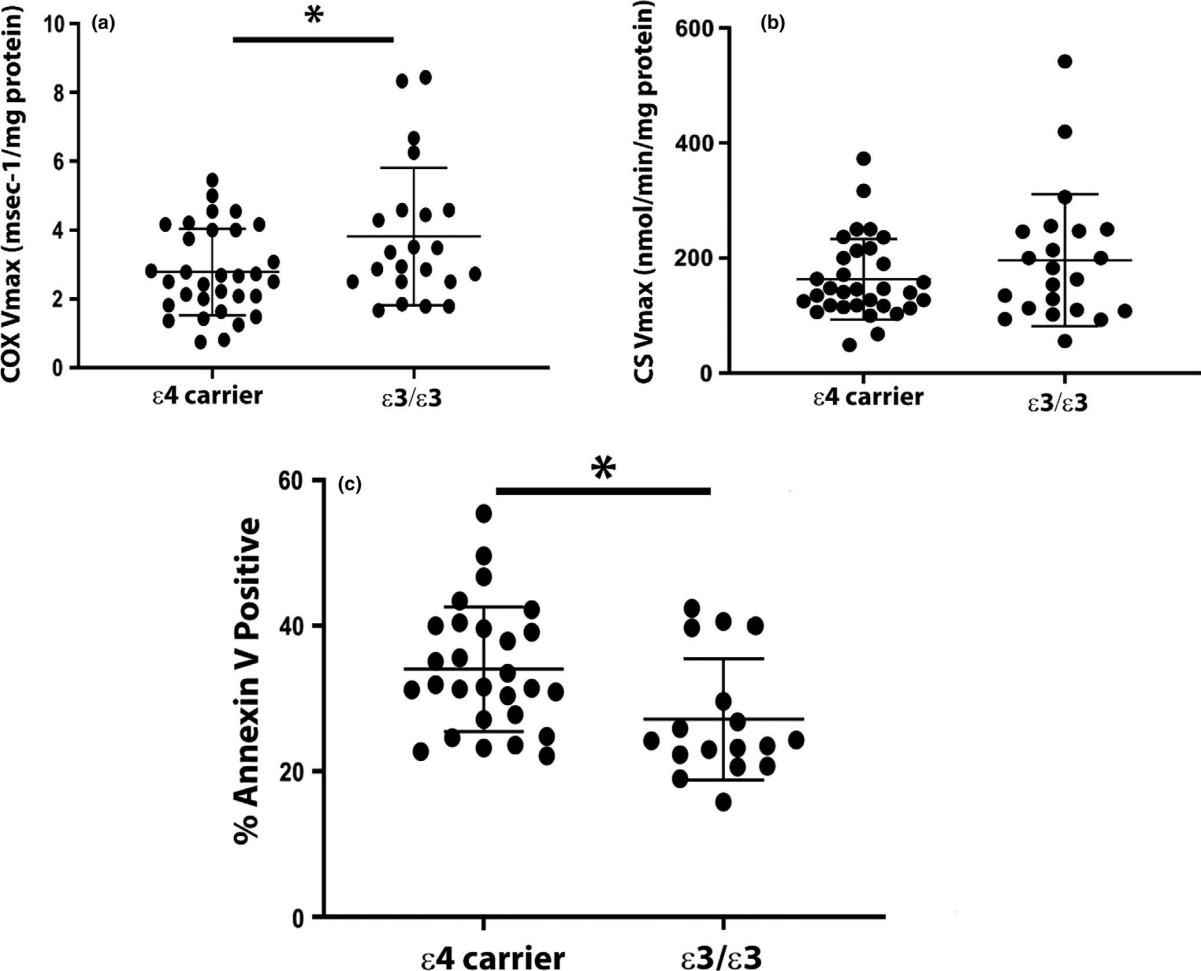
Fresh platelet and lymphocyte biomarkers. Platelet mitochondria were isolated and enzyme Vmax activities measured as described in materials and methods. (a) COX Vmax by *APOE* genotype (*ε4* carriers vs *ε3*/*ε3*). (b) CS Vmax by *APOE* genotype (*ε4* carriers vs *ε3*/*ε3*) * indicates *p* < 0.05, ** indicates *p* < 0.01. Data are shown as mean ± SEM. (c) Lymphocytes were isolated and stained for Annexin V as described in materials and methods. % lymphocytes positive for Annexin V by *APOE* genotype (*ε4* carriers vs *ε3*/*ε3*). * indicates *p* < 0.05

Table 1 provides subject demographics. Table 2 summarizes overall medication use. As we found in a prior study (Wilkins et al., 2017), *APOE ε4* carriers have lower platelet mitochondrial COX Vmax activities when compared to non‐carriers (*ε3*/*ε3*) (Figure 1). Unlike the prior study, we found no change in CS Vmax with *APOE* genotype. Table 2 summarizes enzyme Vmax findings by individual genotypes (heterozygotes versus homozygotes).

**TABLE 1:** Demographics. Systemic biomarker *p* values for APOE ε4 carriers versus non‐carriers are age *p* = 0.98 and sex *p* = 0.99. Human Autopsy Brain Samples p values for APOE ε4 carriers versus non‐carriers are age *p* = 0.38 and sex *p* = 0.61

Systemic biomarker samples		
	Sex (M/F)	Mean age (standard deviation)
APOE ε4 carriers	17/15	73.9 (8.5)
APOE ε4 Non‐carriers	12/10	74.6 (7.4)
APOE ε4/ε4	4/5	71.6 (8.6)
APOE ε3/ε4	13/9	74.2 (8.2)
APOE ε3/ε3	10/9	74.5 (7.1)
APOE ε2 heterozygous	2/2	77.5 (11.6)

Human autopsy brain samples		
	Sex (M/F)	Age
ND	5/2	79.3 (9.3)
AD	5/3	78.9 (9.2)
APOE ε4 carriers	4/3	81.3 (7.7)
APOE ε4 Non‐carriers	6/2	77.1 (9.8)

**TABLE 2:** Medications by group. Values are % of total. Chi‐square *p* = 0.12

Medication classification	Non‐carriers	APOE ε4 carriers
Vitamin/supplement	73.9	100.0
Blood pressure	47.8	53.8
Cholesterol	39.1	46.2
Anti‐depressant	34.8	53.8
Opiod/pain/muscle relaxer	8.7	15.4
Beta‐blocker	17.4	30.8
Anti‐diabetic	13.0	7.7
Dopamine agonist	4.3	3.8
Thyroid	17.4	30.8
Cholinesterase inhibitor	26.1	30.7
NMDA receptor antagonist	4.3	11.5
Cholinesterase inhitibor+NMDA antagonist	65.2	53.8

Lymphocytes from *APOE ε4* carriers and non‐carriers showed similar levels of mitochondrial superoxide production (MitoSox), mitochondrial membrane potential (JC1), and mitochondrial number (MitoTracker). These data are summarized in Table S3. The MitoSox and MitoTracker measurements, though, featured a high standard deviation. We did, however, observe an increase in apoptotic lymphocytes from *APOE ε4* carriers versus *ε3*/*ε3* non‐carriers (Figure 1).

**FIGURE 2:**
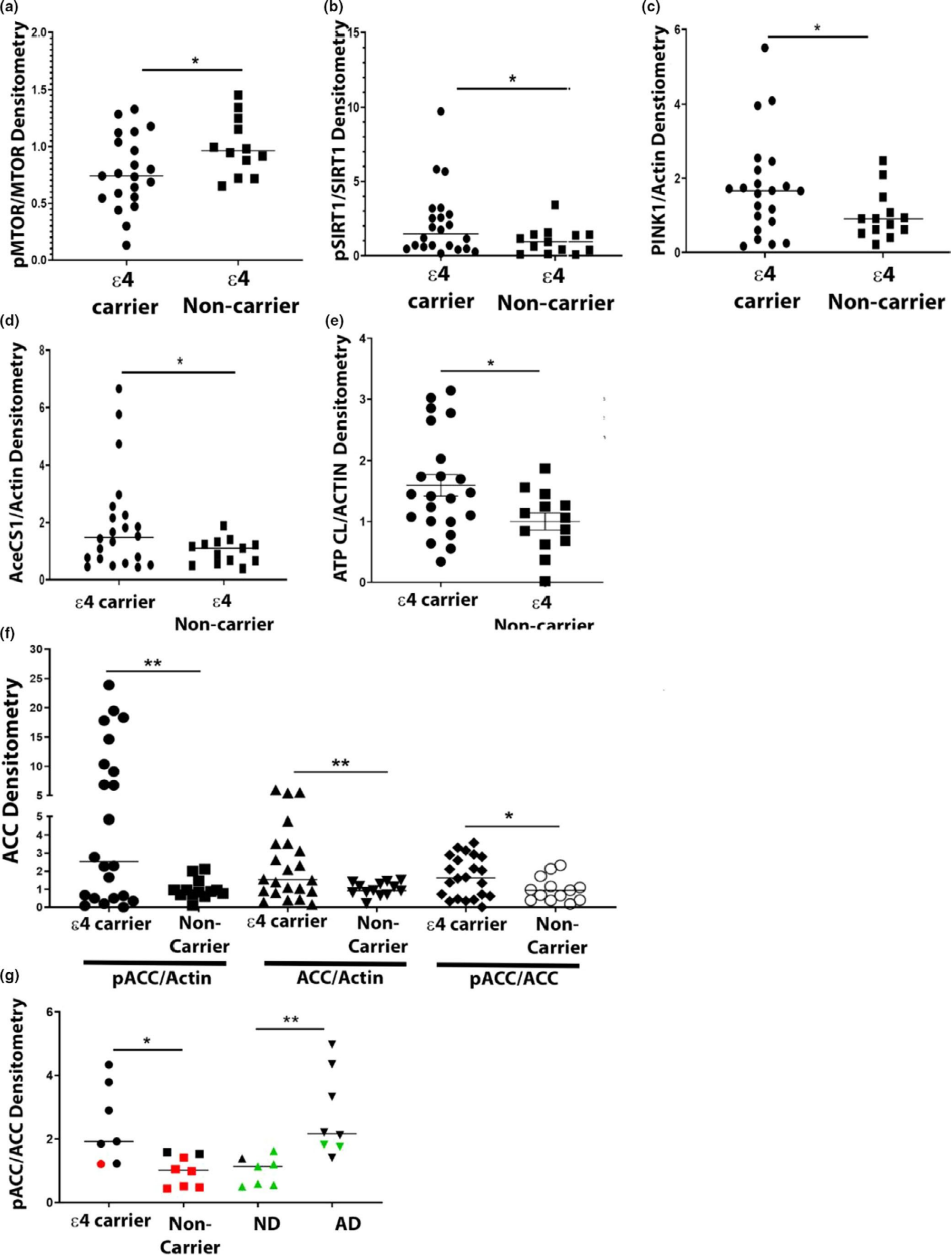
Cultured lymphocyte energy sensing pathway protein expression. Lymphocytes were lysed and assayed for protein expression as described in materials and methods. (a) Lymphocyte PINK/ACTIN densitometry by *APOE* genotype. (b) Lymphocyte pSIRT1/SIRT1 densitometry by *APOE* genotype. (c) Lymphocyte pMTOR/MTOR densitometry by *APOE* genotype. (d) Lymphocyte AceCS1/ACTIN densitometry by *APOE* genotype. (e) Lymphocyte ATP CL/ACTIN densitometry by *APOE* genotype. (f) Lymphocyte ACC densitometry by *APOE* genotype. (g) ACC densitometry in autopsied human brain samples by *APOE* genotype or diagnosis. Red indicates ND subjects when data are separated by *APOE* genotype. Green indicates *APOE ε4* Non‐Carriers when data are separated by diagnosis. * indicates *p* < 0.05, ** indicates *p* < 0.01. Data are shown as mean ± SEM

To facilitate lymphocyte protein expression assays, we expanded them in culture. Levels of PINK1 (PTEN‐induced kinase 1), a protein involved in mitophagy, were higher in *APOE ε4* carrier lymphocytes. mTOR (mammalian target of rapamycin) and SIRT1 (Sirtuin 1), which play a role in energy sensing, were different between groups. Specifically, mTOR phosphorylation decreased while SIRT1 phosphorylation increased in *APOE ε4* carrier lymphocytes. We also observed changes in the lipid synthesis pathway. AceCSI (acetyl‐CoA synthase 1) and ATP CL (ATP citrate lyase) increased, and total ACC and ACC (acetyl‐CoA carboxylase) phosphorylation increased (Figure 2). For data summarizing, all proteins examined see Table S4.

The change in ACC phosphorylation appeared especially robust, which prompted us to examine this parameter in the superior frontal gyrus of post‐mortem AD and non‐demented human brains. *APOE ε4* carrier post‐mortem human brain samples showed increased pACC regardless of whether the subject donor carried an AD diagnosis (Figure 2).

**FIGURE 3:**
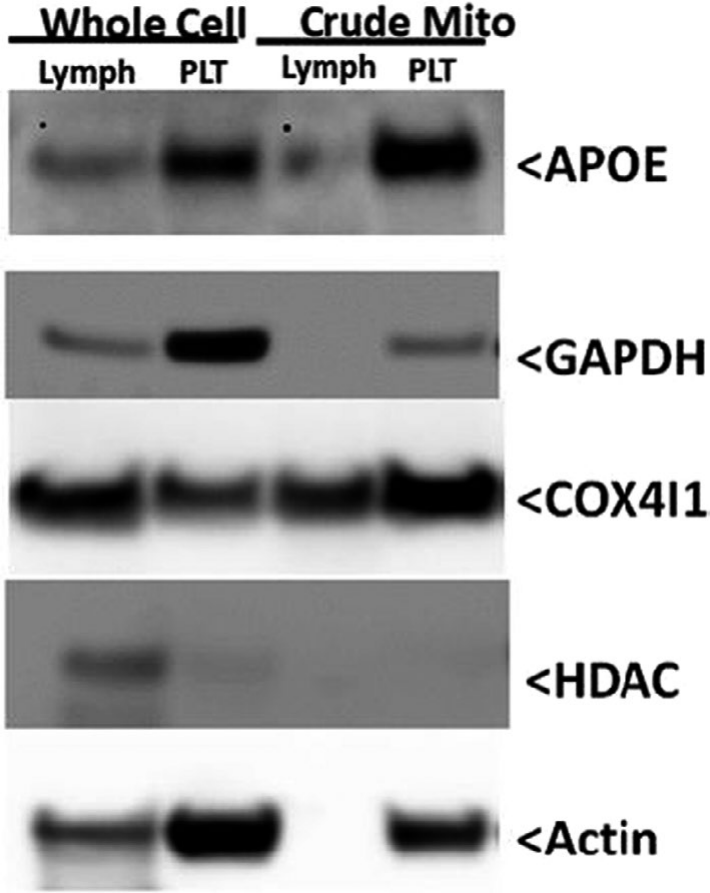
Platelets and lymphocytes express APOE. Lymphocytes and platelets were isolated from whole blood and lysed. Lymphocyte and platelets were also subjected to mitochondrial isolation using nitrogen cavitation and differential centrifugation. Western blots show expression of APOE in both cell types with some mitochondrial localization. GAPDH is a cytosolic marker. COX4I1 is a mitochondrial marker. HDAC is a nuclear marker. Actin is a cytoskeletal protein

As *APOE* genotype associated with biochemical differences in both lymphocytes and platelets, we assessed for the presence of APOE protein in both platelets and lymphocytes. Western blots of lymphocyte and platelet lysates showed APOE protein was in fact present in the lysates (Figure 3). APOE protein was also seen in enriched mitochondrial lysates from lymphocytes and platelets, which suggests at least some of the APOE protein observed in the lysates resides intracellularly (Figure 3).

**FIGURE 4:**
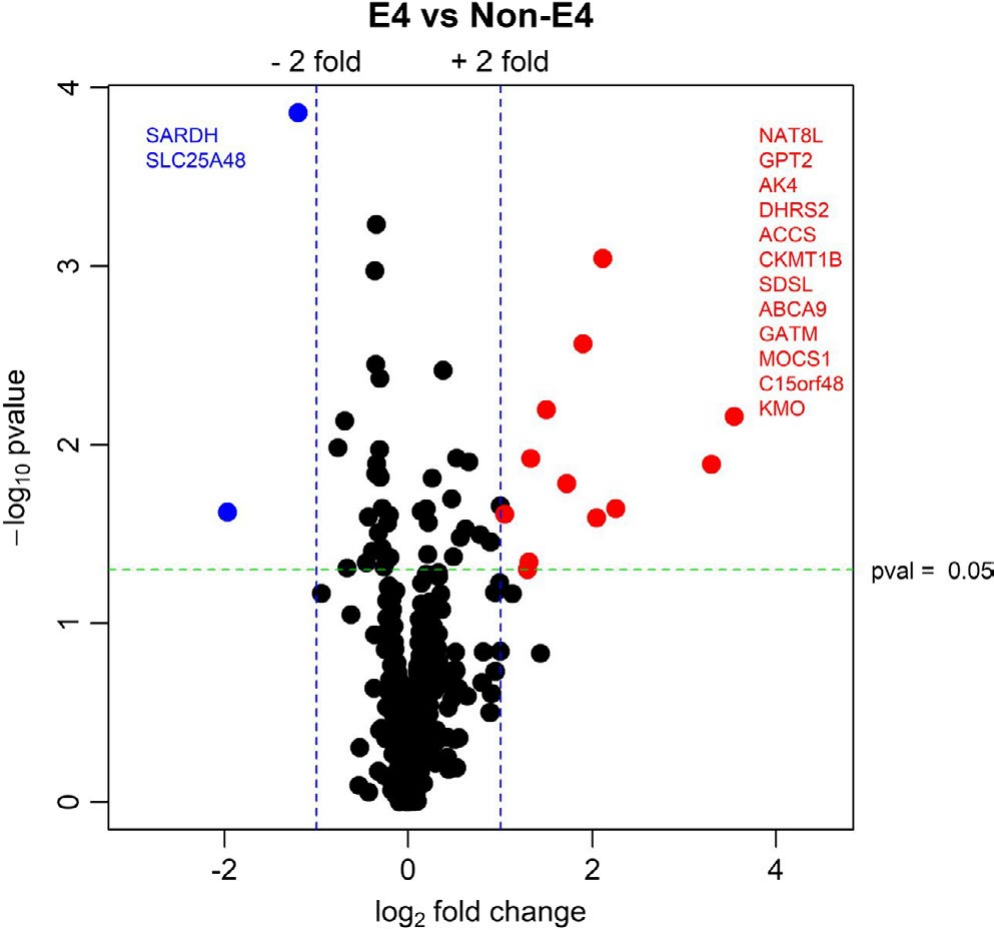
Cultured lymphocyte RNAseq MitoCarta3.0 analysis. RNA was isolated from lymphocytes using phenol/chloroform extraction, and RNAseq was completed. Log fold changes for genes were inputted into the MitoCara3.0 database. Volcano plot by *APOE* genotype of MitoCarta3.0 gene analysis. Red indicates upregulated genes. Blue indicates downregulated genes. Significant gene names are identified with text

**FIGURE 5:**
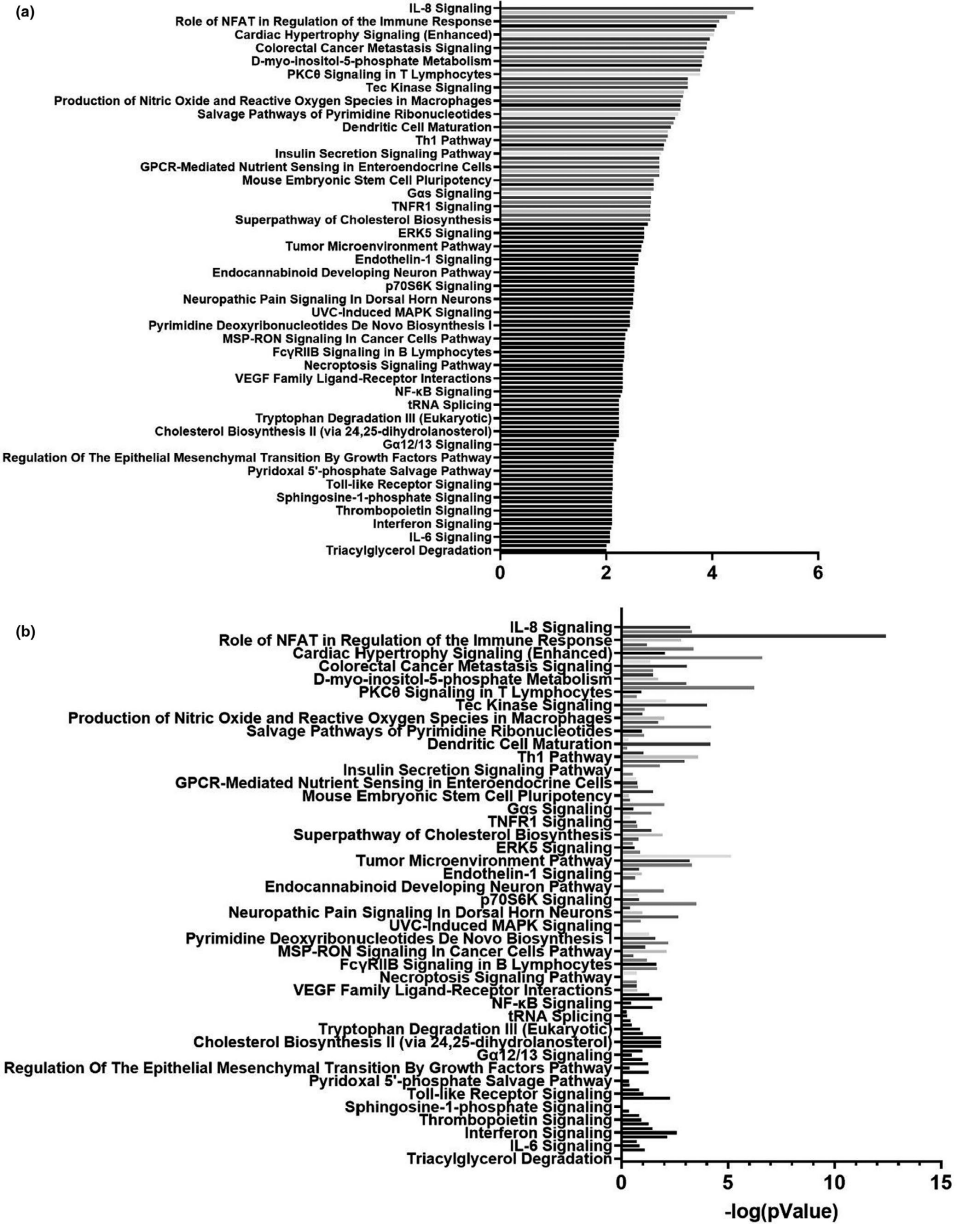
Cultured lymphocyte RNAseq IPA analysis upregulated pathways. RNA was isolated from lymphocytes using phenol/chloroform extraction and RNAseq was completed. Log fold changes for genes were inputted into the IPA database. (a) IPA analysis by *APOE* genotype *z* scores for individual pathways. (b) *p* values of pathways from (a)

**FIGURE 6:**
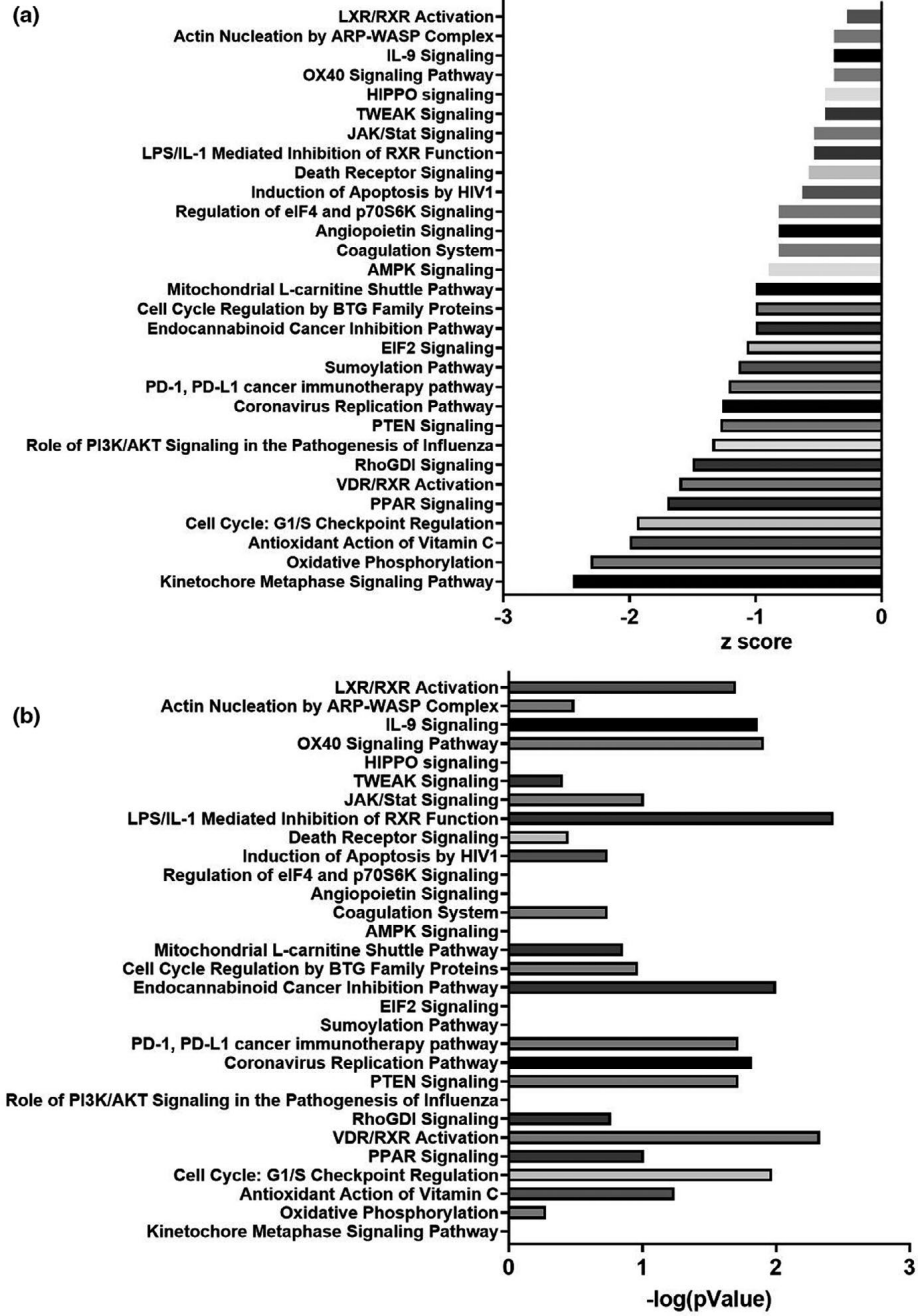
Cultured lymphocyte RNAseq IPA analysis downregulated pathways. RNA was isolated from lymphocytes using phenol/chloroform extraction and RNAseq was completed. Log fold changes for genes were inputted into the IPA database. (a) IPA analysis by *APOE* genotype *z* scores for individual pathways. (b) *p* values of pathways from (a)

Lymphocyte cultures were subjected to RNAseq. Two significant gene expression changes were observed. Transcript levels for Sphingolipid Transporter 3 (SPNS3), a lipid transport molecule, and Integrin Subunit Alpha 1 (ITGA1), a cell adhesion molecule, were lower in AD *APOE ε4* carrier lymphocytes (Table S5). Pathway analysis using the MitoCarta3.0 gene set showed changes to fatty acid metabolism (both degradation and biosynthesis), apoptosis, metabolic pathways (numerous amino acid synthesis pathways), and NFκB (nuclear factor kappa B) signaling (Figure 4, Table 3 and Table S6). Ingenuity pathway analysis (IPA) revealed upregulated pathways in AD *APOE ε4* carrier lymphocytes were more numerous and included metabolic (cholesterol biosynthesis and insulin secretion) and inflammatory pathways (Figure 5). Downregulation of cell cycle, mitochondrial L‐carnitine transport, oxidative phosphorylation, and multiple immune‐mediated pathways in AD *APOE ε4* carrier lymphocytes were observed with IPA analysis (Figure 6).

**TABLE 3:** KEGG analysis of MitoCarta3.0 genes by ApoE genotype

Pathway	DE	P.DE
Biosynthesis of cofactors	7	7.39E‐07
Metabolic pathways	16	5.57E‐06
Fatty acid metabolism	4	4.02E‐05
Apoptosis—multiple species	3	0.000173
Glycine, serine, and threonine metabolism	3	0.000338
Fatty acid degradation	3	0.000391
Arginine and proline metabolism	3	0.000695
Apoptosis	4	0.001151
Adipocytokine signaling pathway	3	0.001678
Fatty acid biosynthesis	2	0.001714
PPAR signaling pathway	3	0.002215
Alanine, aspartate, and glutamate metabolism	2	0.007163
Purine metabolism	3	0.009952
Valine, leucine, and isoleucine biosynthesis	1	0.013799
Aminoacyl‐tRNA biosynthesis	2	0.021688
p53 signaling pathway	2	0.026179
Platinum drug resistance	2	0.026179
Biosynthesis of amino acids	2	0.027527
Colorectal cancer	2	0.035421
Thermogenesis	3	0.04473
NF‐kappa B signaling pathway	2	0.049969

Note

The table includes pathways with *p* values (P.DE) below 0.05.

Abbreviations: DE, differential expression; P.DE, *p* value of differential expression.

Kyoto Encyclopedia of Genes and Genomes (KEGG) pathway analysis corroborated the IPA. Implicated KEGG pathways are highlighted in Figures S1–S6. Cell signaling pathways were altered including HIF1α (hypoxia‐inducible factor 1 alpha) and PI3K/AKT (phosphoinositide 3‐kinase/protein kinase B). Pathways that promote anaerobic metabolism, drive angiogenesis, and respond to intermittent fasting were upregulated in AD *APOE ε4* carrier lymphocytes. Within the PI3K/AKT pathway genes which respond to and initiate inflammation (cytokines, Toll‐like receptors), hypoxia, cell cycle, and apoptosis/cell survival were upregulated in AD *APOE ε4* carrier lymphocytes. Inflammatory pathways were largely upregulated in AD *APOE ε4* carrier lymphocytes and included NFκB and chemokine/cytokine pathways. Tables S7–S10 depict top pathway changes across IPA, KEGG, GO (gene ontology), GSEA (Gene Set Enrichment Analysis), and MitoCarta3.0 analysis. Comparison of results from multiple pathway analysis programs is important to ensure agreement between statistical analysis methods. Interestingly, IPA showed activation of dementia and AD‐related pathways in AD *APOE ε4* carrier lymphocytes (Table S7 and S8). For example, *APOE ε4* carrier lymphocytes demonstrated increased expression of the matrix metalloprotease 9 (MMP9) pathway components, which participates in amyloid clearance, and increased amyloid precursor protein (APP) pathway expression.

## 4 DISCUSSION

Here, we independently corroborate a smaller study (Wilkins et al., 2017) that found lower platelet COX activity in women with AD and an *APOE ε4* allele, versus those without an *APOE ε4* allele. We now further extend this finding to men with AD and an *APOE ε4* allele. We did not confirm the smaller study's finding of lower platelet CS Vmax activities in *APOE ε4* carriers. Although differences in particular medications occurred between individuals, by studying the impact of the APOE genotype within an AD population, we avoided the major confound of having most participants in one group using cholinesterase inhibitors and no participants in the other group using cholinesterase inhibitors. Further, as APOE effects in this study were demonstrated in blood cells, they are unlikely to represent confounds of neurodegeneration or the classic AD‐related protein aggregations.

COX Vmax is lower in AD subjects, a finding reported in brain, fibroblasts, and blood cells (Swerdlow et al., 1998; Wilkins et al., 2017). Mechanistic studies show that COX is assembled differently in AD subjects, which may contribute to Vmax deficits (Parker & Parks, 1995). Additional studies associate the COX deficit with changes to its mRNA expression within brain (Simonian & Hyman, 1994). Lower COX activity in AD subjects is at least partly accounted for by mitochondrial DNA, either through inherited variants or somatic mutations (Swerdlow et al., 1997). Deficits in COX functionality will lead to bioenergetic stress including changes in redox balance and ATP production (Silva et al., 2013). As a systemic biomarker, reduced platelet COX Vmax correlates strongly with reduced brain glucose metabolism (Mosconi et al., 2017). Future studies should leverage this systemic biomarker to understand the origins of bioenergetic stress observed in AD.

Lymphocyte apoptosis can be attributed to “neglect” or loss of extrinsic signals, a process that occurs through mitochondrial energy failure and the loss of anaplerosis (Vander Heiden et al., 1999; Whetton & Dexter, 1983). Bioenergetic stress, as a consequence of reduced glucose metabolism, may play a role in lymphocyte apoptosis (Rathmell et al., 2000; Vander Heiden et al., 2001). Our Annexin V staining and RNAseq data support changes to metabolism and apoptotic signaling in AD *APOE ε4* carrier lymphocytes. Our overall findings suggest increased lymphocyte apoptosis may reflect a consequence of bioenergetic stress.

A previous study claimed SIRT1 phosphorylation at the site we interrogated reflects SIRT1 activation (Sasaki et al., 2008). SIRT1 regulates chromatin remodeling, allowing for gene expression changes that adapt to stress (Bosch‐Presegue & Vaquero, 2014). SIRT1 functions to alter cell metabolism including glycolysis flux, lipid homeostasis, insulin secretion, and inflammation. Energy stress activates SIRT1, which essentially serves as a stress response master regulator (Bosch‐Presegue & Vaquero, 2014).

mTOR promotes cell growth. It activates under anabolic conditions that coincide with energy‐sufficient states and deactivates under catabolic conditions of energy stress. Serine 2448 phosphorylation levels positively correlate with mTOR activity and suggest a downstream assembly of mTORC2 and mTORC1, protein complexes implicated in cell metabolic regulation (Copp et al., 2009; Jhanwar‐Uniyal et al., 2019). Decreased mTOR 2448 phosphorylation in *APOE ε4* carrier lymphocytes suggests that allele shifts the anabolic–catabolic balance to a more catabolic setting.

Cells experiencing catabolic shifts typically increase autophagy, a process of internal digestion that replenishes raw molecular materials. PINK1 helps mediate autophagy, and increased PINK1 suggests increased mitophagy/autophagy flux (Nguyen et al., 2016). Furthermore, mTOR modulates autophagy through unc‐51 like kinase 1 (ULK1) as inhibition of mTOR during nutrient starvation leads to activation of ULK1 and autophagy (Dunlop & Tee, 2014). In this case, elevated PINK1 and reduced mTOR activation in AD *APOE ε4* carrier lymphocytes suggest some degree of cell‐level energy stress in the *APOE ε4* carriers.

The most robust finding in lymphocytes was the increase in pACC and ACC expression by *APOE* genotype. ACC is an enzyme which converts acetyl‐CoA into malonyl CoA through carboxylation, which represents an early integral step in fatty acid synthesis. ACC phosphorylation inhibits its activity and turns off lipid biosynthesis. To understand whether this change was specific, we also examined ACC expression and phosphorylation in human post‐mortem brain samples. We found that pACC levels also increased in brains from AD and *APOE ε4* carriers.

AceCS1 is a cytosolic enzyme that catalyzes the conversion of acetate and CoA to acetyl‐CoA, where it enters lipid synthesis. SIRT1 reportedly regulates its activity (Hallows et al., 2006). ATP CL converts citrate to acetyl‐CoA and oxaloacetate, and links carbohydrate and fatty acid metabolism. Both ATP CL and AceCS1 expression are higher in *APOE ε4* carriers, which could represent a cause or consequence of the observed ACC changes. AceCS1 and ATP CL could potentially increase whether an ACC‐mediated reduction in lipid biosynthesis leads to a secondary increase in acetyl‐CoA. Based on this pattern of observations, investigating acetyl‐CoA and its up/downstream metabolites in *APOE ε4* carriers could prove informative. In mice with an *APOE ε4* genotype, adipogenesis and lipogenesis are impaired in adipose tissue (Arbones‐Mainar et al., 2010). Our results are consistent with these findings that APOE ε4 genotype impairs lipogenesis across multiple tissue and cell types.

APOE interacts with multiple pathways. For example, it is notable that *APOE ε4* mediates the downregulation of PPAR and VDR/RXR signaling (Dawson & Xia, 2012). PPARγ suppresses macrophage inflammatory pathways, which is potentially relevant to the observation that inflammatory pathways are upregulated in *APOE ε4* carrier lymphocytes (Chawla, 2010; Moore et al., 2001). In this respect, it is interesting that PPARγ agonists failed to elicit adipogenesis in a study of *APOE ε4* mice, because *APOE ε4* suppressed PPARγ expression in adipose tissues and macrophages (Arbones‐Mainar et al., 2010).

RNAseq data further indicate lymphocytes from *APOE* *ε4* carriers experience metabolic stress. Multiple pathway analyses showed reductions in fatty acid metabolism, metabolite transport, and oxidative phosphorylation. Hypoxic signaling and apoptotic signaling are upregulated. RNAseq data also reveal an overall pro‐inflammatory state in *APOE ε4* carriers, with activation of the NFκB signaling pathway and increased cytokine/chemokine expression.

Our data agree with other studies that find specific APOE‐determined molecular phenotypes extend beyond the brain (Badia et al., 2013; Wilkins et al., 2017). At the very least astrocytes express APOE in the brain, but as lymphocytes and platelets are not generally recognized as APOE producing cell types the question of why APOE‐associated phenotypes exist in these cell populations requires consideration. We did detect APOE protein in platelet and lymphocyte lysates, and our data indicate at least some of that APOE is intracellular. Our data do not address whether platelet and lymphocyte affiliated APOE was imported or locally produced, although since platelets lack nuclei locally generated APOE would need to accumulate either at the megakaryocyte stage or through the translation of persisting *APOE* transcripts. *APOE* isoforms also reside in close linkage dysequilibrium with variants of the Translocase of the Outer Mitochondrial Membrane 40 kD subunit (*TOMM40*) gene (Roses, 2010). *APOE* isoforms could therefore serve as a surrogate for *TOMM40* variants that independently influence mitochondrial function and metabolism. Further, as neurons increase their *APOE* expression under stress conditions, it is worth considering whether other cell types, when stressed, additionally demonstrate this behavior (Aoki et al., 2003; Boschert et al., 1999). Finally, myeloid APOE can alter lymphocyte biology through non‐cell autonomous signaling events (Bonacina et al., 2018). Future studies will need to consider focusing on these potential mechanisms.

Despite years of research, the mechanisms that underlie the AD *APOE* association remain unclear. Our data support the view that bioenergetic metabolism‐related stress may mediate this. As APOE expression occurs beyond the brain and defines specific molecular patterns, it seems reasonable to propose a systemic AD phenotype may exist. This could manifest as subtle metabolic shifts of the type we now demonstrate and could perhaps explain other associations including reported connections between dementia and type II diabetes mellitus (Arnold et al., 2018). This study also argues peripheral metabolism biomarkers may reflect brain metabolism. If further validated, peripheral biomarkers like the ones we now show, or those developed by others (Chacko et al., 2014), could provide valuable insight into AD biology, suggest treatment strategies, and monitor target engagement in clinical trials.

## CONFLICT OF INTEREST

The authors declare that they have no conflict of interest.

## AUTHORS’ CONTRIBUTIONS

HMW and RHS conceptualized and obtained funding for the work. HMW developed and performed assays and wrote the manuscript. SJM, SJK, XW, and BWM assisted with data collection. RB, AMB, HA, ES, and JMB recruited and consented participants. MH oversees the KU ADRC Brain Bank. PC performed RNAseq data and pathway analysis. RHS obtained HSC approval and edited the manuscript.

## Ethics approval and consent to participate

The Kansas University Medical Center Human Subjects Committee (KUMC HSC) approved all human subject participation and all participants provided informed consent prior to enrolling. This study was conducted in accordance with the Code of Ethics of the World Medical Association (the Declaration of Helsinki).

## Supporting information

Fig S1‐S6

Table S1‐S10

## Data Availability

The datasets generated and/or analyzed during the current study are not publicly available due to restrictions with our HSC approval but are available from the corresponding author on reasonable request.

## References

[acel13356-bib-0001] Aoki, K. , Uchihara, T. , Sanjo, N. , Nakamura, A. , Ikeda, K. , Tsuchiya, K. , & Wakayama, Y. (2003). Increased expression of neuronal apolipoprotein E in human brain with cerebral infarction. Stroke, 34(4), 875–880. 10.1161/01.STR.0000064320.73388.C6 12649507

[acel13356-bib-0002] Arbones‐Mainar, J. M. , Johnson, L. A. , Altenburg, M. K. , Kim, H. S. , & Maeda, N. (2010). Impaired adipogenic response to thiazolidinediones in mice expressing human apolipoproteinE4. The FASEB Journal, 24(10), 3809–3818. 10.1096/fj.10-159517 20501792 PMC2996914

[acel13356-bib-0003] Arnold, S. E. , Arvanitakis, Z. , Macauley‐Rambach, S. L. , Koenig, A. M. , Wang, H.‐Y. , Ahima, R. S. , Craft, S. , Gandy, S. , Buettner, C. , Stoeckel, L. E. , Holtzman, D. M. , & Nathan, D. M. (2018). Brain insulin resistance in type 2 diabetes and Alzheimer disease: Concepts and conundrums. Nature Reviews Neurology, 14(3), 168–181. 10.1038/nrneurol.2017.185 29377010 PMC6098968

[acel13356-bib-0004] Badia, M. C. , Lloret, A. , Giraldo, E. , Dasi, F. , Olaso, G. , Alonso, M. D. , & Vina, J. (2013). Lymphocytes from young healthy persons carrying the ApoE4 allele overexpress stress‐related proteins involved in the pathophysiology of Alzheimer's disease. Journal of Alzheimer's Disease, 33(1), 77–83. 10.3233/JAD-2012-120973 22914590

[acel13356-bib-0005] Bonacina, F. , Coe, D. , Wang, G. , Longhi, M. P. , Baragetti, A. , Moregola, A. , Garlaschelli, K. , Uboldi, P. , Pellegatta, F. , Grigore, L. , Da Dalt, L. , Annoni, A. , Gregori, S. , Xiao, Q. , Caruso, D. , Mitro, N. , Catapano, A. L. , Marelli‐Berg, F. M. , & Norata, G. D. (2018). Myeloid apolipoprotein E controls dendritic cell antigen presentation and T cell activation. Nature Communications, 9(1), 3083. 10.1038/s41467-018-05322-1 PMC6079066 30082772

[acel13356-bib-0006] Boschert, U. , Merlo‐Pich, E. , Higgins, G. , Roses, A. D. , & Catsicas, S. (1999). Apolipoprotein E expression by neurons surviving excitotoxic stress. Neurobiology of Diseases, 6(6), 508–514. 10.1006/nbdi.1999.0251 10600406

[acel13356-bib-0007] Bosch‐Presegue, L. , & Vaquero, A. (2014). Sirtuins in stress response: Guardians of the genome. Oncogene, 33(29), 3764–3775. 10.1038/onc.2013.344 23995787

[acel13356-bib-0008] Cavedo, E. , Lista, S. , Rojkova, K. , Chiesa, P. A. , Houot, M. , Brueggen, K. , Blautzik, J. , Bokde, A. L. W. , Dubois, B. , Barkhof, F. , Pouwels, P. J. W. , Teipel, S. , Hampel, H. , & Alzheimer Precision Medicine Initiative (APMI) (2017). Disrupted white matter structural networks in healthy older adult APOE epsilon4 carriers – An international multicenter DTI study. Neuroscience, 357, 119–133. 10.1016/j.neuroscience.2017.05.048 28596117

[acel13356-bib-0009] Chacko, B. K. , Kramer, P. A. , Ravi, S. , Benavides, G. A. , Mitchell, T. , Dranka, B. P. , Ferrick, D. , Singal, A. K. , Ballinger, S. W. , Bailey, S. M. , Hardy, R. W. , Zhang, J. , Zhi, D. , & Darley‐Usmar, V. M. (2014). The Bioenergetic Health Index: a new concept in mitochondrial translational research. Clinical Science, 127(6), 367–373. 10.1042/CS20140101 24895057 PMC4202728

[acel13356-bib-0010] Chawla, A. (2010). Control of macrophage activation and function by PPARs. Circulation Research, 106(10), 1559–1569. 10.1161/CIRCRESAHA.110.216523 20508200 PMC2897247

[acel13356-bib-0011] Copp, J. , Manning, G. , & Hunter, T. (2009). TORC‐specific phosphorylation of mammalian target of rapamycin (mTOR): Phospho‐Ser2481 is a marker for intact mTOR signaling complex 2. Cancer Research, 69(5), 1821–1827. 10.1158/0008-5472.CAN-08-3014 19244117 PMC2652681

[acel13356-bib-0012] Dafnis, I. , Argyri, L. , Sagnou, M. , Tzinia, A. , Tsilibary, E. C. , Stratikos, E. , & Chroni, A. (2016). The ability of apolipoprotein E fragments to promote intraneuronal accumulation of amyloid beta peptide 42 is both isoform and size‐specific. Scientific Reports, 6, 30654. 10.1038/srep30654 27476701 PMC4967930

[acel13356-bib-0013] Dawson, M. I. , & Xia, Z. (2012). The retinoid X receptors and their ligands. Biochimica Et Biophysica Acta, 1821(1), 21–56. 10.1016/j.bbalip.2011.09.014 22020178 PMC4097889

[acel13356-bib-0014] Dunlop, E. A. , & Tee, A. R. (2014). mTOR and autophagy: A dynamic relationship governed by nutrients and energy. Seminars in Cell & Developmental Biology, 36, 121–129. 10.1016/j.semcdb.2014.08.006 25158238

[acel13356-bib-0015] Evans, S. , Dowell, N. G. , Tabet, N. , Tofts, P. S. , King, S. L. , & Rusted, J. M. (2014). Cognitive and neural signatures of the APOE E4 allele in mid‐aged adults. Neurobiology of Aging, 35(7), 1615–1623. 10.1016/j.neurobiolaging.2014.01.145 24582638 PMC4001126

[acel13356-bib-0016] Filippini, N. , Ebmeier, K. P. , MacIntosh, B. J. , Trachtenberg, A. J. , Frisoni, G. B. , Wilcock, G. K. , Beckmann, C. F. , Smith, S. M. , Matthews, P. M. , & Mackay, C. E. (2011). Differential effects of the APOE genotype on brain function across the lifespan. NeuroImage, 54(1), 602–610. 10.1016/j.neuroimage.2010.08.009 20705142

[acel13356-bib-0017] Hallows, W. C. , Lee, S. , & Denu, J. M. (2006). Sirtuins deacetylate and activate mammalian acetyl‐CoA synthetases. Proceedings of the National Academy of Sciences of the United States of America, 103(27), 10230–10235. 10.1073/pnas.0604392103 16790548 PMC1480596

[acel13356-bib-0018] Jhanwar‐Uniyal, M. , Wainwright, J. V. , Mohan, A. L. , Tobias, M. E. , Murali, R. , Gandhi, C. D. , & Schmidt, M. H. (2019). Diverse signaling mechanisms of mTOR complexes: mTORC1 and mTORC2 in forming a formidable relationship. Advances in Biological Regulation, 72, 51–62. 10.1016/j.jbior.2019.03.003 31010692

[acel13356-bib-0019] Kim, E. , Park, M. , Jeong, J. , Kim, H. , Lee, S. K. , Lee, E. , Oh, B. H. , & Namkoong, K. (2016). Cholinesterase inhibitor donepezil increases mitochondrial biogenesis through AMP‐activated protein kinase in the hippocampus. Neuropsychobiology, 73(2), 81–91. 10.1159/000441522 27002982

[acel13356-bib-0020] Langmead, B. , & Salzberg, S. L. (2012). Fast gapped‐read alignment with Bowtie 2. Nature methods, 9(4), 357. 10.1038/nmeth.1923 22388286 PMC3322381

[acel13356-bib-0021] Li, B. , & Dewey, C. N. (2011). RSEM: accurate transcript quantification from RNA‐Seq data with or without a reference genome. BMC bioinformatics, 12(1), 1–16. 10.1186/1471-2105-12-323 21816040 PMC3163565

[acel13356-bib-0022] Liberzon, A. , Subramanian, A. , Pinchback, R. , Thorvaldsdottir, H. , Tamayo, P. , & Mesirov, J. P. (2011). Molecular signatures database (MSigDB) 3.0. Bioinformatics, 27(12), 1739–1740. 10.1093/bioinformatics/btr260 21546393 PMC3106198

[acel13356-bib-0023] Luo, W. , & Brouwer, C. (2013). Pathview: An R/Bioconductor package for pathway‐based data integration and visualization. Bioinformatics, 29(14), 1830–1831. 10.1093/bioinformatics/btt285 23740750 PMC3702256

[acel13356-bib-0024] Luo, W. , Friedman, M. S. , Shedden, K. , Hankenson, K. D. , & Woolf, P. J. (2009). GAGE: Generally applicable gene set enrichment for pathway analysis. BMC Bioinformatics, 10, 161. 10.1186/1471-2105-10-161 19473525 PMC2696452

[acel13356-bib-0025] Mahley, R. W. , & Huang, Y. (2012). Apolipoprotein e sets the stage: Response to injury triggers neuropathology. Neuron, 76(5), 871–885. 10.1016/j.neuron.2012.11.020 23217737 PMC4891195

[acel13356-bib-0026] McKhann, G. M. , Knopman, D. S. , Chertkow, H. , Hyman, B. T. , Jack, C. R. , Kawas, C. H. , Klunk, W. E. , Koroshetz, W. J. , Manly, J. J. , Mayeux, R. , Mohs, R. C. , Morris, J. C. , Rossor, M. N. , Scheltens, P. , Carrillo, M. C. , Thies, B. , Weintraub, S. , & Phelps, C. H. (2011). The diagnosis of dementia due to Alzheimer's disease: Recommendations from the National Institute on Aging‐Alzheimer's Association workgroups on diagnostic guidelines for Alzheimer's disease. Alzheimer's & Dementia: the Journal of the Alzheimer's Association, 7(3), 263–269. 10.1016/j.jalz.2011.03.005 PMC3312024 21514250

[acel13356-bib-0027] Moore, K. J. , Rosen, E. D. , Fitzgerald, M. L. , Randow, F. , Andersson, L. P. , Altshuler, D. , Milstone, D. S. , Mortensen, R. M. , Spiegelman, B. M. , & Freeman, M. W. (2001). The role of PPAR‐gamma in macrophage differentiation and cholesterol uptake. Nature Medicine, 7(1), 41–47. 10.1038/83328 11135614

[acel13356-bib-0028] Morris, J. K. , Uy, R. A. Z. , Vidoni, E. D. , Wilkins, H. M. , Archer, A. E. , Thyfault, J. P. , Miles, J. M. , & Burns, J. M. (2017). Effect of APOE epsilon4 genotype on metabolic biomarkers in aging and Alzheimer's disease. Journal of Alzheimer's Disease, 58(4), 1129–1135. 10.3233/JAD-170148 PMC5776708 28550261

[acel13356-bib-0029] Mosconi, L. , Berti, V. , Guyara‐Quinn, C. , McHugh, P. , Petrongolo, G. , Osorio, R. S. , Connaughty, C. , Pupi, A. , Vallabhajosula, S. , Isaacson, R. S. , de Leon, M. J. , Swerdlow, R. H. , & Brinton, R. D. (2017). Perimenopause and emergence of an Alzheimer's bioenergetic phenotype in brain and periphery. PLoS One, 12(10), e0185926. 10.1371/journal.pone.0185926 29016679 PMC5634623

[acel13356-bib-0030] Mosconi, L. , Nacmias, B. , Sorbi, S. , De Cristofaro, M. T. , Fayazz, M. , Tedde, A. , Bracco, L. , Herholz, K. , & Pupi, A. (2004). Brain metabolic decreases related to the dose of the ApoE e4 allele in Alzheimer's disease. Journal of Neurology, Neurosurgery and Psychiatry, 75(3), 370–376. 10.1136/jnnp.2003.014993 PMC1738980 14966149

[acel13356-bib-0031] Mouchard, A. , Boutonnet, M. C. , Mazzocco, C. , Biendon, N. , Macrez, N. , & Neuro, C. E. B. N. N. (2019). ApoE‐fragment/Abeta heteromers in the brain of patients with Alzheimer's disease. Scientific Reports, 9(1), 3989. 10.1038/s41598-019-40438-4 30850702 PMC6408522

[acel13356-bib-0032] Nguyen, T. N. , Padman, B. S. , & Lazarou, M. (2016). Deciphering the molecular signals of PINK1/Parkin mitophagy. Trends in Cell Biology, 26(10), 733–744. 10.1016/j.tcb.2016.05.008 27291334

[acel13356-bib-0033] Parker, W. D. Jr , & Parks, J. K. (1995). Cytochrome c oxidase in Alzheimer's disease brain: Purification and characterization. Neurology, 45(3 Pt 1), 482–486. 10.1212/wnl.45.3.482 7898701

[acel13356-bib-0034] Rathmell, J. C. , Vander Heiden, M. G. , Harris, M. H. , Frauwirth, K. A. , & Thompson, C. B. (2000). In the absence of extrinsic signals, nutrient utilization by lymphocytes is insufficient to maintain either cell size or viability. Molecular Cell, 6(3), 683–692. 10.1016/s1097-2765(00)00066-6 11030347

[acel13356-bib-0035] Robinson, M. D. , McCarthy, D. J. , & Smyth, G. K. (2010). edgeR: a Bioconductor package for differential expression analysis of digital gene expression data. Bioinformatics, 26(1), 139–140. 10.1093/bioinformatics/btp616 19910308 PMC2796818

[acel13356-bib-0036] Roses, A. D. (2010). An inherited variable poly‐T repeat genotype in TOMM40 in Alzheimer disease. Archives of Neurology, 67(5), 536–541. 10.1001/archneurol.2010.88 20457951 PMC3140162

[acel13356-bib-0037] Sasaki, T. , Maier, B. , Koclega, K. D. , Chruszcz, M. , Gluba, W. , Stukenberg, P. T. , Minor, W. , & Scrable, H. (2008). Phosphorylation regulates SIRT1 function. PLoS One, 3(12), e4020. 10.1371/journal.pone.0004020 19107194 PMC2602979

[acel13356-bib-0038] Silva, D. F. , Selfridge, J. E. , Lu, J. , E, L. , Roy, N. , Hutfles, L. , Burns, J. M. , Michaelis, E. K. , Yan, S. , Cardoso, S. M. , & Swerdlow, R. H. (2013). Bioenergetic flux, mitochondrial mass and mitochondrial morphology dynamics in AD and MCI cybrid cell lines. Human Molecular Genetics, 22(19), 3931–3946. 10.1093/hmg/ddt247 23740939 PMC3888119

[acel13356-bib-0039] Simonian, N. A. , & Hyman, B. T. (1994). Functional alterations in Alzheimer's disease: Selective loss of mitochondrial‐encoded cytochrome oxidase mRNA in the hippocampal formation. Journal of Neuropathology and Experimental Neurology, 53(5), 508–512. 10.1097/00005072-199409000-00010 8083692

[acel13356-bib-0040] Subramanian, A. , Tamayo, P. , Mootha, V. K. , Mukherjee, S. , Ebert, B. L. , Gillette, M. A. , Paulovich, A. , Pomeroy, S. L. , Golub, T. R. , Lander, E. S. , & Mesirov, J. P. (2005). Gene set enrichment analysis: a knowledge‐based approach for interpreting genome‐wide expression profiles. Proceedings of the National Academy of Sciences of the United States of America, 102(43), 15545–15550. 10.1073/pnas.0506580102 16199517 PMC1239896

[acel13356-bib-0041] Swerdlow, R. H. , Parks, J. K. , Cassarino, D. S. , Maguire, D. J. , Maguire, R. S. , Bennett, J. P. , Davis, R. E. , & Parker, W. D. (1997). Cybrids in Alzheimer's disease: A cellular model of the disease? Neurology, 49(4), 918–925. 10.1212/wnl.49.4.918 9339668

[acel13356-bib-0042] Swerdlow, R. H. , Parks, J. K. , Cassarino, D. S. , Trimmer, P. A. , Miller, S. W. , Maguire, D. J. , Sheehan, J. P. , Maguire, R. S. , Pattee, G. , Juel, V. C. , Phillips, L. H. , Tuttle, J. B. , Bennett, J. P. , Davis, R. E. , & Parker, W. D. (1998). Mitochondria in sporadic amyotrophic lateral sclerosis. Experimental Neurology, 153(1), 135–142. 10.1006/exnr.1998.6866 9743575

[acel13356-bib-0043] Vander Heiden, M. G. , Chandel, N. S. , Schumacker, P. T. , & Thompson, C. B. (1999). Bcl‐xL prevents cell death following growth factor withdrawal by facilitating mitochondrial ATP/ADP exchange. Molecular Cell, 3(2), 159–167. 10.1016/s1097-2765(00)80307-x 10078199

[acel13356-bib-0044] Vander Heiden, M. G. , Plas, D. R. , Rathmell, J. C. , Fox, C. J. , Harris, M. H. , & Thompson, C. B. (2001). Growth factors can influence cell growth and survival through effects on glucose metabolism. Molecular and Cellular Biology, 21(17), 5899–5912. 10.1128/mcb.21.17.5899-5912.2001 11486029 PMC87309

[acel13356-bib-0045] Wellnitz, S. , Friedlein, A. , Bonanni, C. , Anquez, V. , Goepfert, F. , Loetscher, H. , Adessi, C. , & Czech, C. (2005). A 13 kDa carboxy‐terminal fragment of ApoE stabilizes Abeta hexamers. Journal of Neurochemistry, 94(5), 1351–1360. 10.1111/j.1471-4159.2005.03295.x 16011742

[acel13356-bib-0046] Whetton, A. D. , & Dexter, T. M. (1983). Effect of haematopoietic cell growth factor on intracellular ATP levels. Nature, 303(5918), 629–631. 10.1038/303629a0 6855907

[acel13356-bib-0047] Wilkins, H. M. , Koppel, S. J. , Bothwell, R. , Mahnken, J. , Burns, J. M. , & Swerdlow, R. H. (2017). Platelet cytochrome oxidase and citrate synthase activities in APOE epsilon4 carrier and non‐carrier Alzheimer's disease patients. Redox Biology, 12, 828–832. 10.1016/j.redox.2017.04.010 28448944 PMC5406545

[acel13356-bib-0048] Yin, J. , Reiman, E. M. , Beach, T. G. , Serrano, G. E. , Sabbagh, M. N. , Nielsen, M. , Caselli, R. J. , & Shi, J. (2020). Effect of ApoE isoforms on mitochondria in Alzheimer disease. Neurology, 94(23), e2404–e2411. 10.1212/WNL.0000000000009582 32457210 PMC7455369

