# Supplementary material for: Bioenergetic and inflammatory systemic phenotypes in Alzheimer’s disease APOE ε4‐carriers
Source: Aging Cell. 2021 May 3;20(5):e13356. doi: 10.1111/acel.13356 (PMC8135087; doi:10.1111/acel.13356)
Supplement: Supplementary file 1 — Fig S1‐S6 [file ACEL-20-e13356-s001.pptx]

## Slide 1
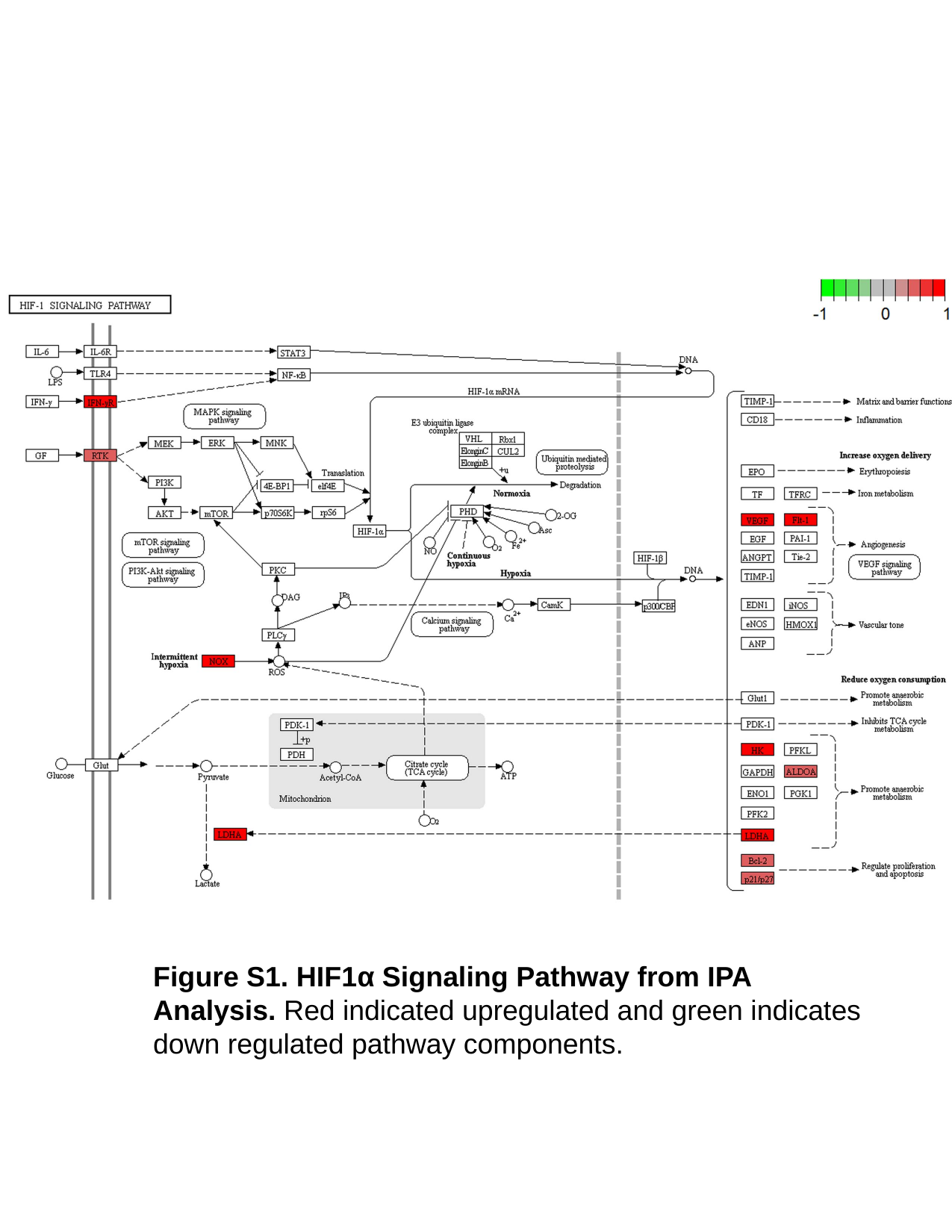

Figure S1. HIF1α Signaling Pathway from IPA Analysis. Red indicated upregulated and green indicates down regulated pathway components.

## Slide 2
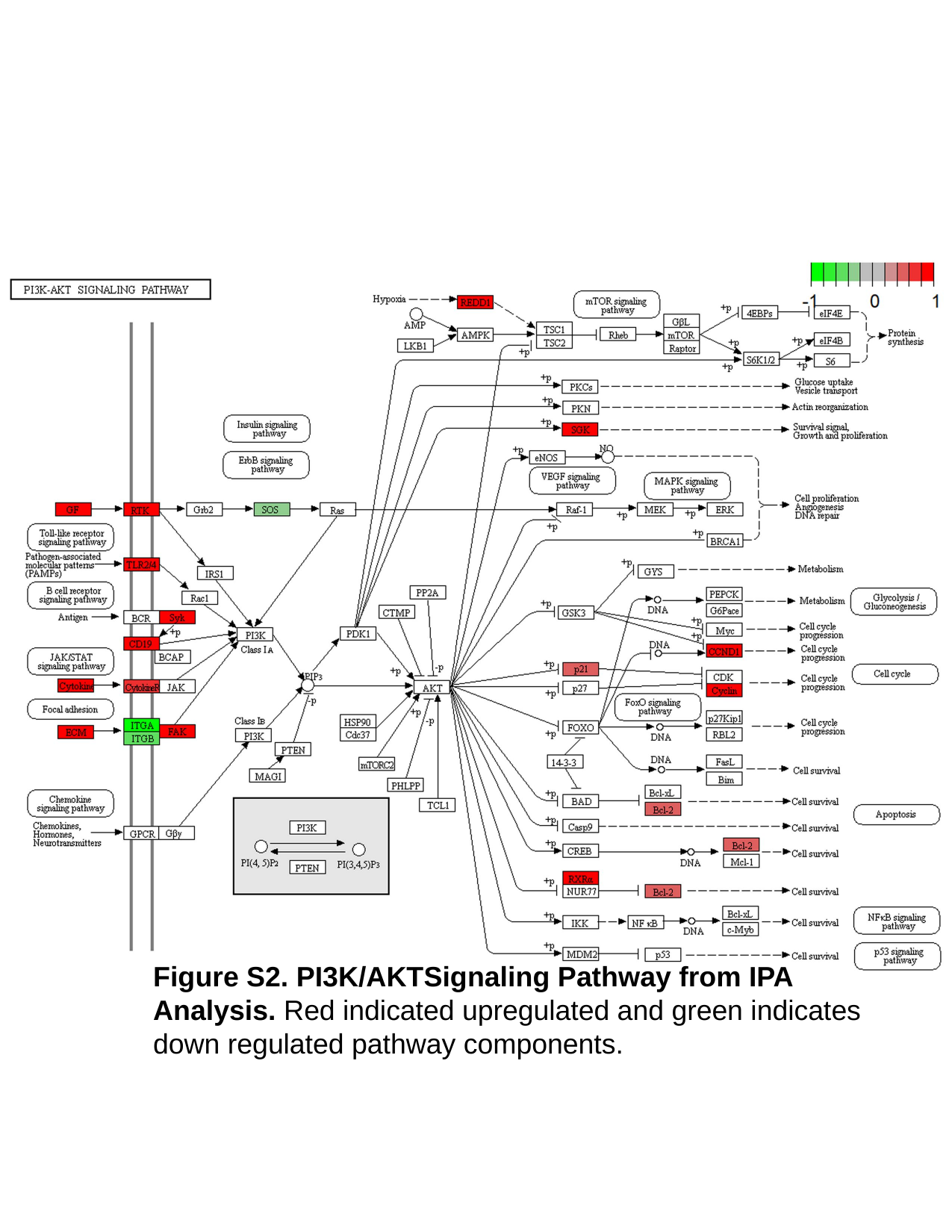

Figure S2. PI3K/AKTSignaling Pathway from IPA Analysis. Red indicated upregulated and green indicates down regulated pathway components.

## Slide 3
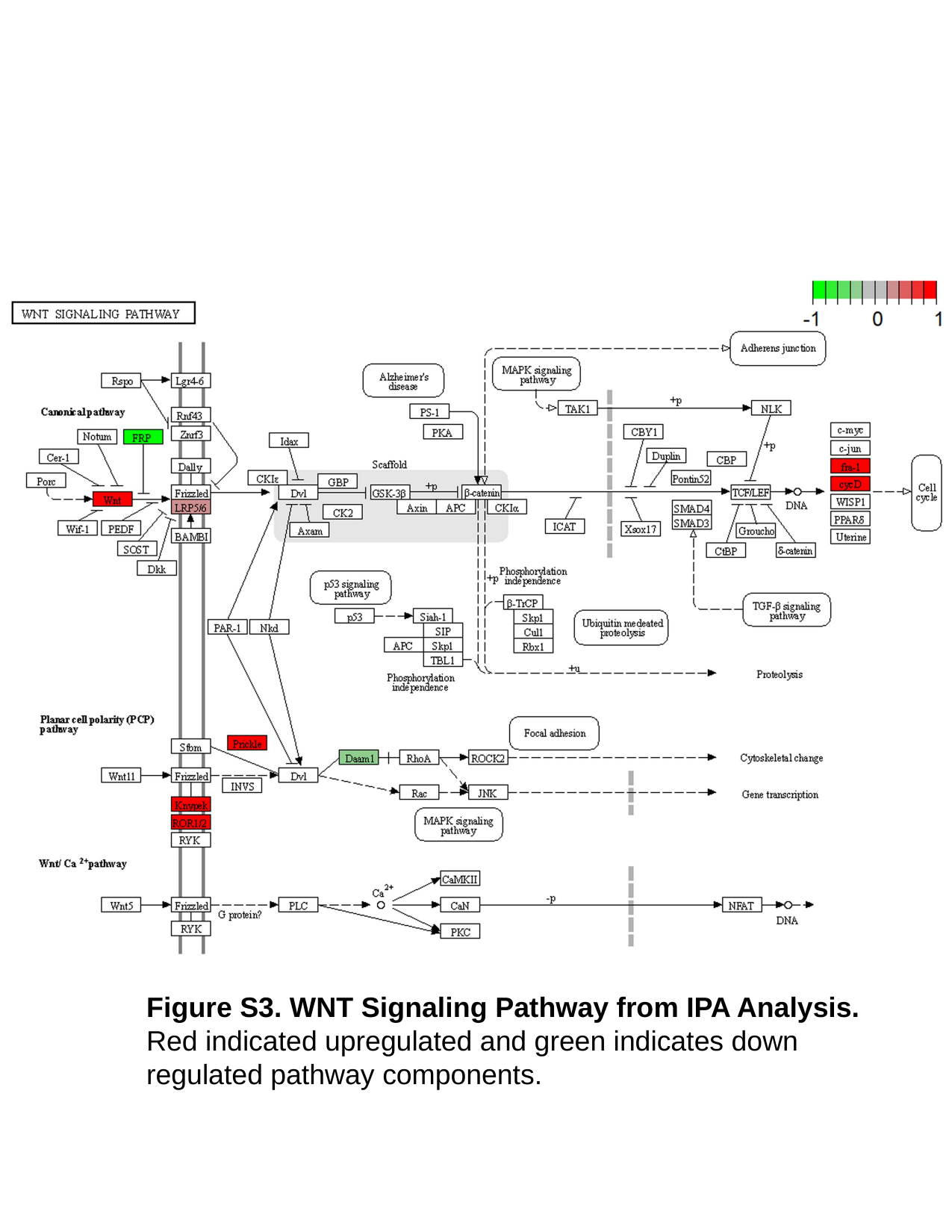

Figure S3. WNT Signaling Pathway from IPA Analysis. Red indicated upregulated and green indicates down regulated pathway components.

## Slide 4
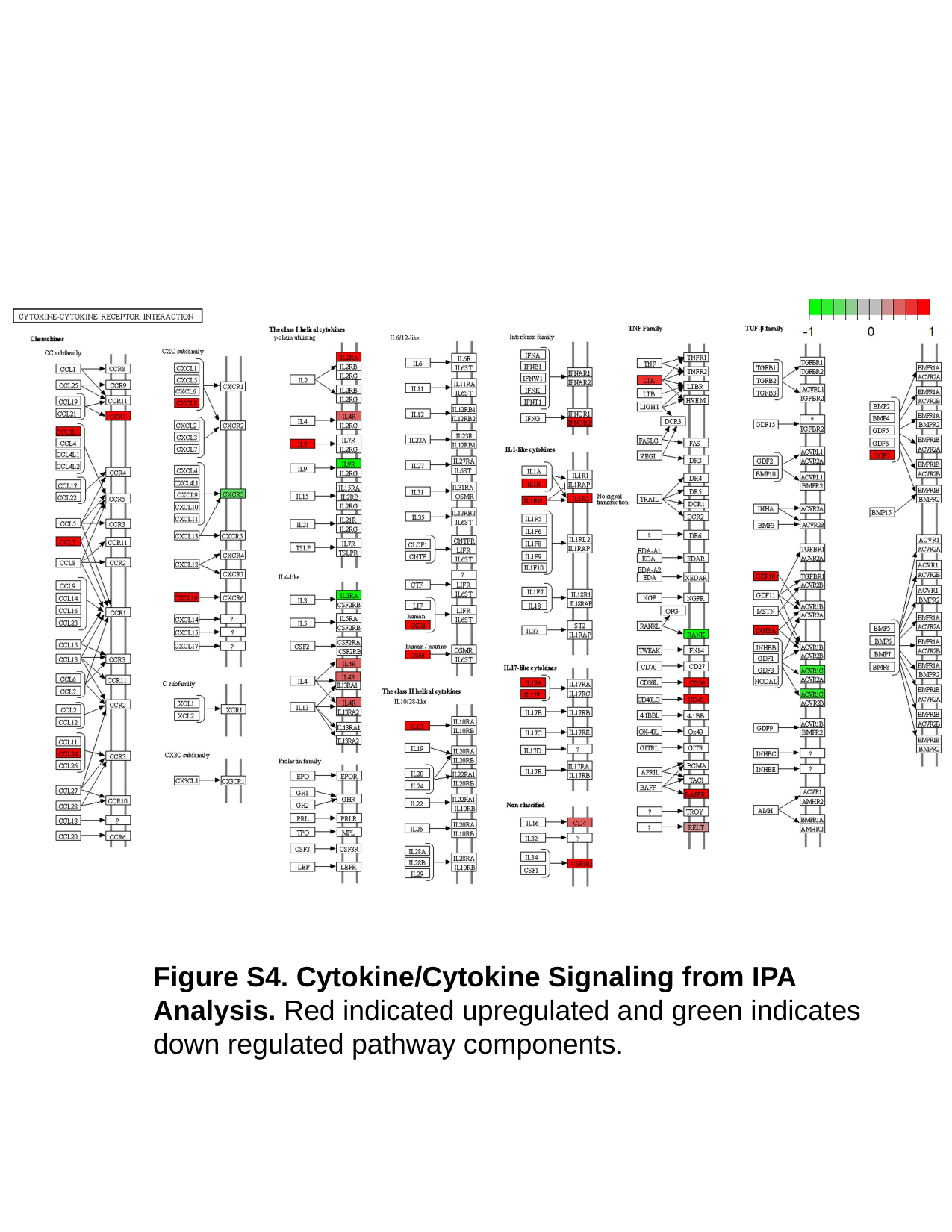

Figure S4. Cytokine/Cytokine Signaling from IPA Analysis. Red indicated upregulated and green indicates down regulated pathway components.

## Slide 5
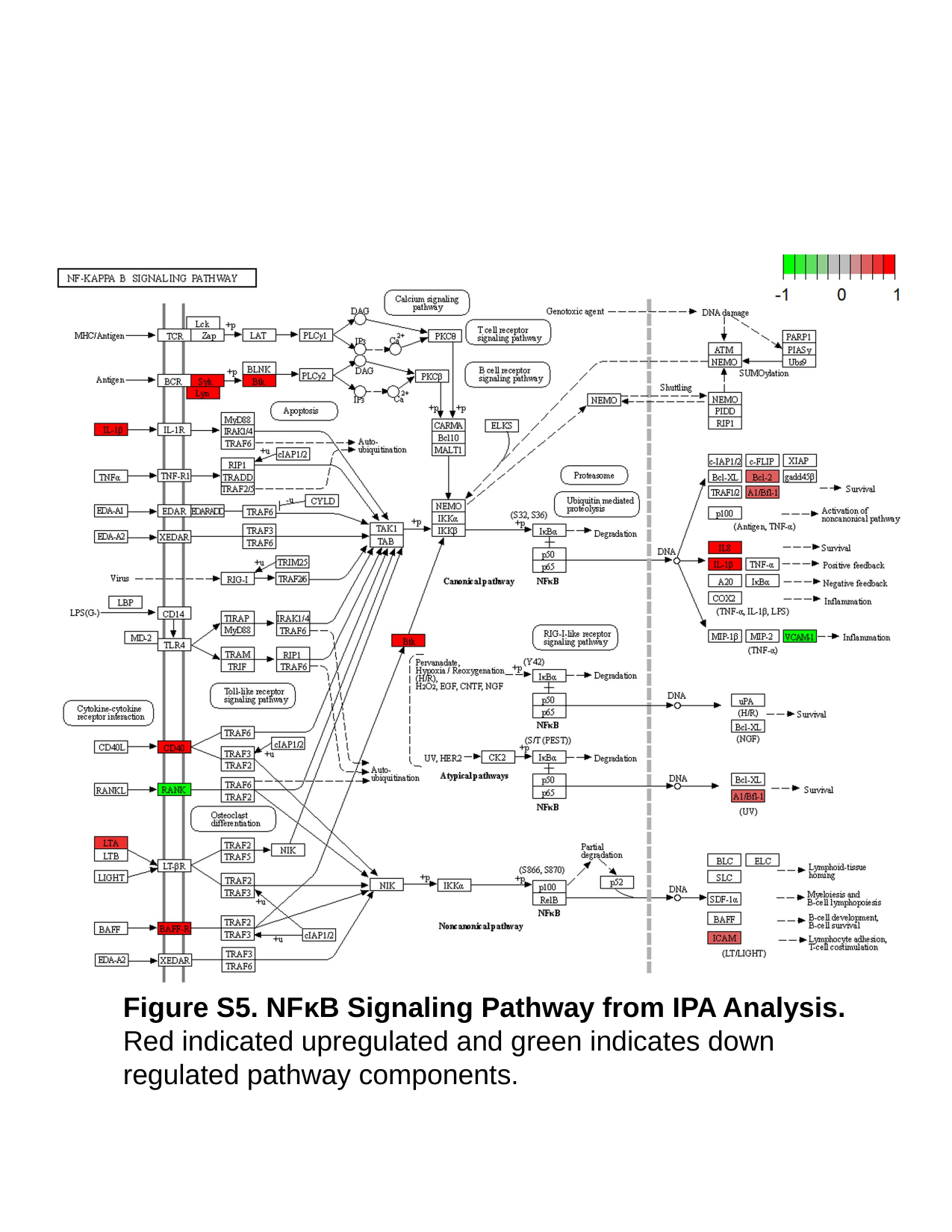

Figure S5. NFκB Signaling Pathway from IPA Analysis. Red indicated upregulated and green indicates down regulated pathway components.

## Slide 6
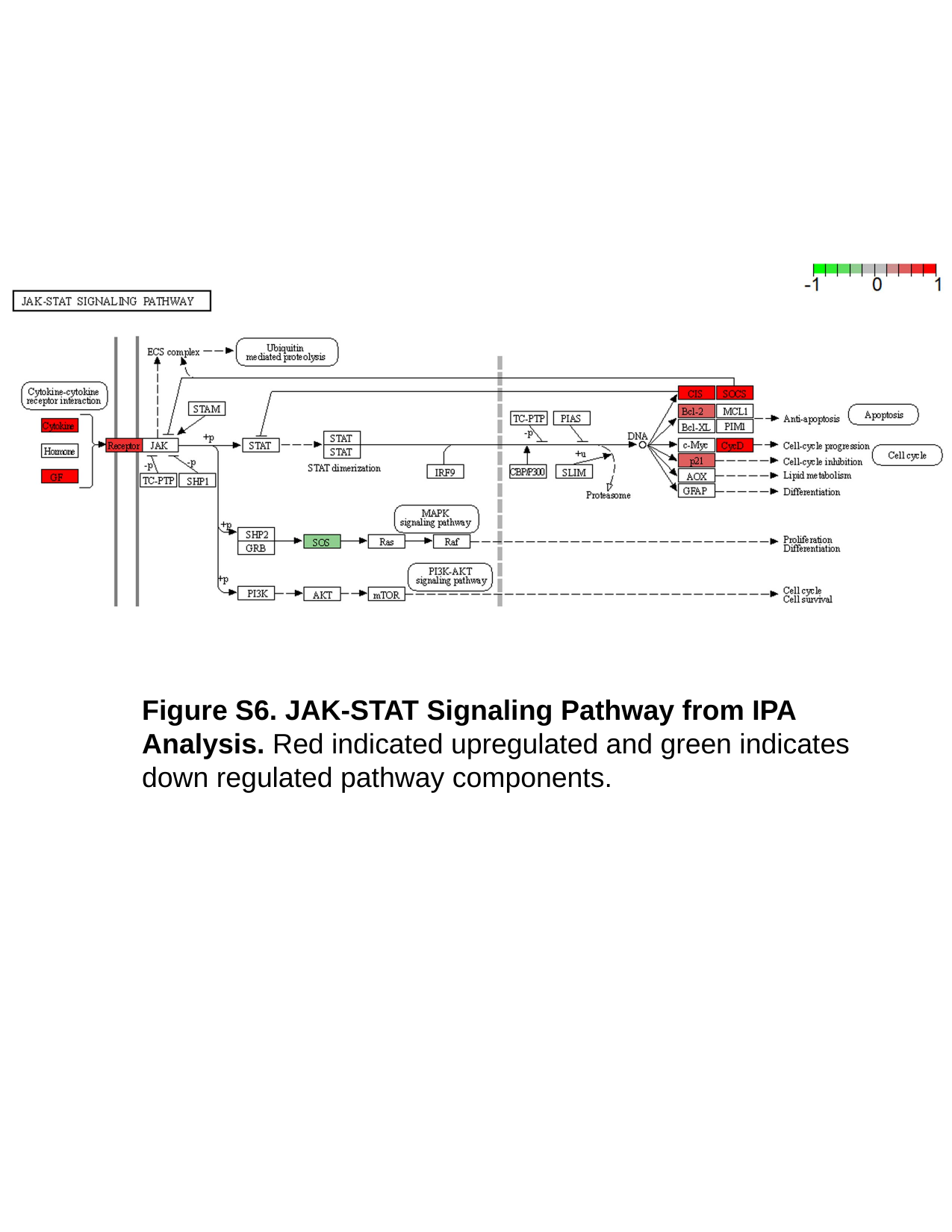

Figure S6. JAK-STAT Signaling Pathway from IPA Analysis. Red indicated upregulated and green indicates down regulated pathway components.

## Slide 7
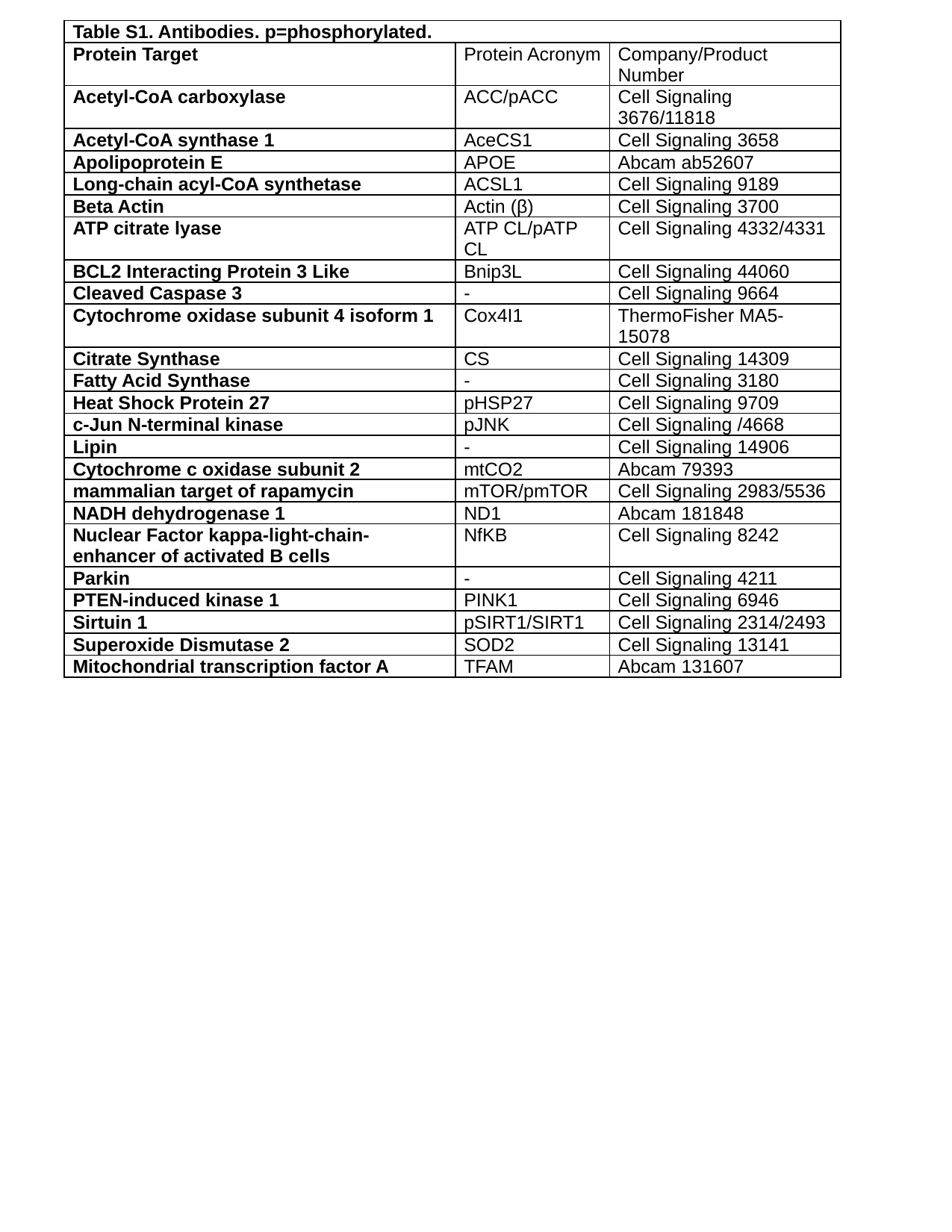

| Table S1. Antibodies. p=phosphorylated. | | |
| --- | --- | --- |
| Protein Target | Protein Acronym | Company/Product Number |
| Acetyl-CoA carboxylase | ACC/pACC | Cell Signaling 3676/11818 |
| Acetyl-CoA synthase 1 | AceCS1 | Cell Signaling 3658 |
| Apolipoprotein E | APOE | Abcam ab52607 |
| Long-chain acyl-CoA synthetase | ACSL1 | Cell Signaling 9189 |
| Beta Actin | Actin (β) | Cell Signaling 3700 |
| ATP citrate lyase | ATP CL/pATP CL | Cell Signaling 4332/4331 |
| BCL2 Interacting Protein 3 Like | Bnip3L | Cell Signaling 44060 |
| Cleaved Caspase 3 | - | Cell Signaling 9664 |
| Cytochrome oxidase subunit 4 isoform 1 | Cox4I1 | ThermoFisher MA5-15078 |
| Citrate Synthase | CS | Cell Signaling 14309 |
| Fatty Acid Synthase | - | Cell Signaling 3180 |
| Heat Shock Protein 27 | pHSP27 | Cell Signaling 9709 |
| c-Jun N-terminal kinase | pJNK | Cell Signaling /4668 |
| Lipin | - | Cell Signaling 14906 |
| Cytochrome c oxidase subunit 2 | mtCO2 | Abcam 79393 |
| mammalian target of rapamycin | mTOR/pmTOR | Cell Signaling 2983/5536 |
| NADH dehydrogenase 1 | ND1 | Abcam 181848 |
| Nuclear Factor kappa-light-chain-enhancer of activated B cells | NfKB | Cell Signaling 8242 |
| Parkin | - | Cell Signaling 4211 |
| PTEN-induced kinase 1 | PINK1 | Cell Signaling 6946 |
| Sirtuin 1 | pSIRT1/SIRT1 | Cell Signaling 2314/2493 |
| Superoxide Dismutase 2 | SOD2 | Cell Signaling 13141 |
| Mitochondrial transcription factor A | TFAM | Abcam 131607 |

## Slide 8
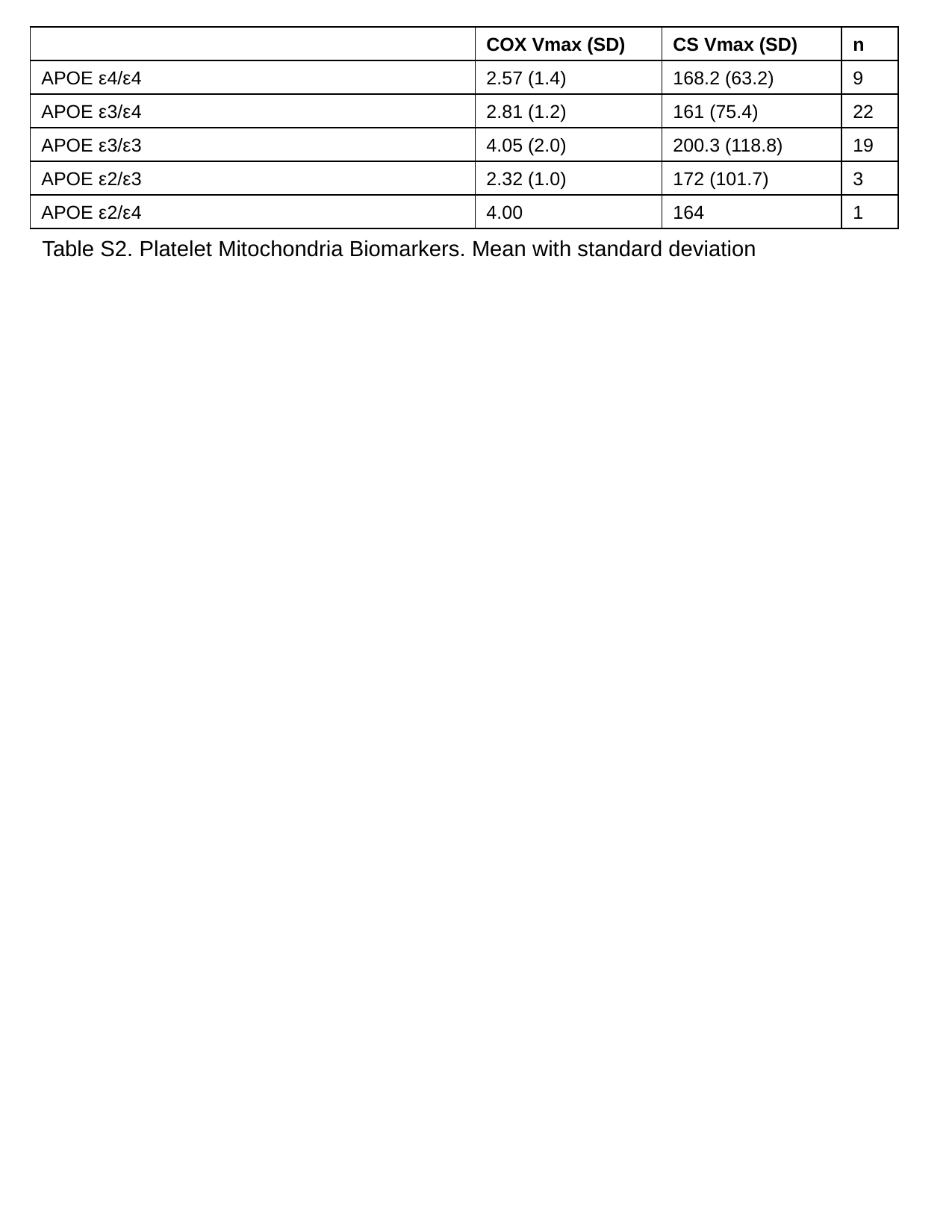

| | COX Vmax (SD) | CS Vmax (SD) | n |
| --- | --- | --- | --- |
| APOE ε4/ε4 | 2.57 (1.4) | 168.2 (63.2) | 9 |
| APOE ε3/ε4 | 2.81 (1.2) | 161 (75.4) | 22 |
| APOE ε3/ε3 | 4.05 (2.0) | 200.3 (118.8) | 19 |
| APOE ε2/ε3 | 2.32 (1.0) | 172 (101.7) | 3 |
| APOE ε2/ε4 | 4.00 | 164 | 1 |
Table S2. Platelet Mitochondria Biomarkers. Mean with standard deviation

## Slide 9
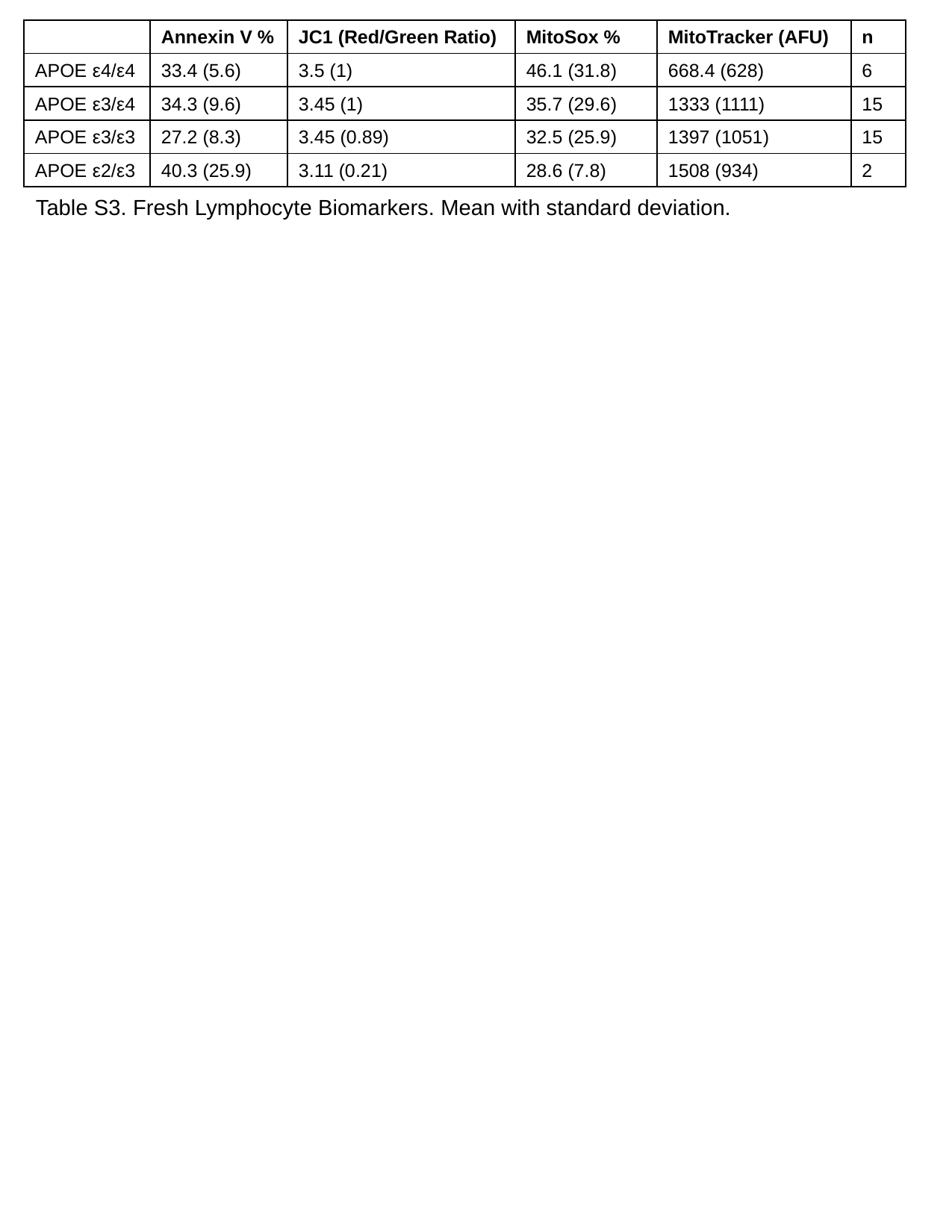

| | Annexin V % | JC1 (Red/Green Ratio) | MitoSox % | MitoTracker (AFU) | n |
| --- | --- | --- | --- | --- | --- |
| APOE ε4/ε4 | 33.4 (5.6) | 3.5 (1) | 46.1 (31.8) | 668.4 (628) | 6 |
| APOE ε3/ε4 | 34.3 (9.6) | 3.45 (1) | 35.7 (29.6) | 1333 (1111) | 15 |
| APOE ε3/ε3 | 27.2 (8.3) | 3.45 (0.89) | 32.5 (25.9) | 1397 (1051) | 15 |
| APOE ε2/ε3 | 40.3 (25.9) | 3.11 (0.21) | 28.6 (7.8) | 1508 (934) | 2 |
Table S3. Fresh Lymphocyte Biomarkers. Mean with standard deviation.

## Slide 10
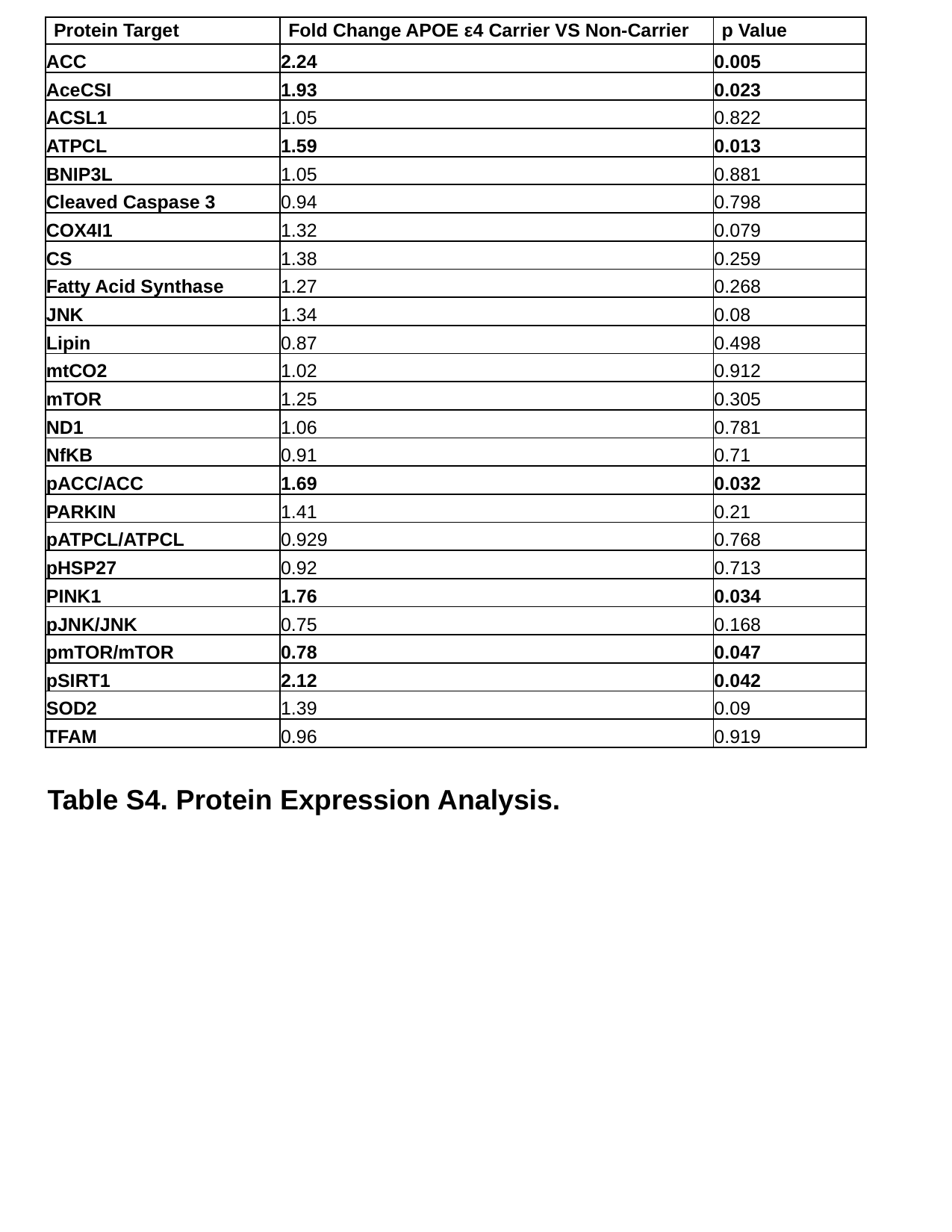

| Protein Target | Fold Change APOE ε4 Carrier VS Non-Carrier | p Value |
| --- | --- | --- |
| ACC | 2.24 | 0.005 |
| AceCSI | 1.93 | 0.023 |
| ACSL1 | 1.05 | 0.822 |
| ATPCL | 1.59 | 0.013 |
| BNIP3L | 1.05 | 0.881 |
| Cleaved Caspase 3 | 0.94 | 0.798 |
| COX4I1 | 1.32 | 0.079 |
| CS | 1.38 | 0.259 |
| Fatty Acid Synthase | 1.27 | 0.268 |
| JNK | 1.34 | 0.08 |
| Lipin | 0.87 | 0.498 |
| mtCO2 | 1.02 | 0.912 |
| mTOR | 1.25 | 0.305 |
| ND1 | 1.06 | 0.781 |
| NfKB | 0.91 | 0.71 |
| pACC/ACC | 1.69 | 0.032 |
| PARKIN | 1.41 | 0.21 |
| pATPCL/ATPCL | 0.929 | 0.768 |
| pHSP27 | 0.92 | 0.713 |
| PINK1 | 1.76 | 0.034 |
| pJNK/JNK | 0.75 | 0.168 |
| pmTOR/mTOR | 0.78 | 0.047 |
| pSIRT1 | 2.12 | 0.042 |
| SOD2 | 1.39 | 0.09 |
| TFAM | 0.96 | 0.919 |
Table S4. Protein Expression Analysis.

## Slide 11
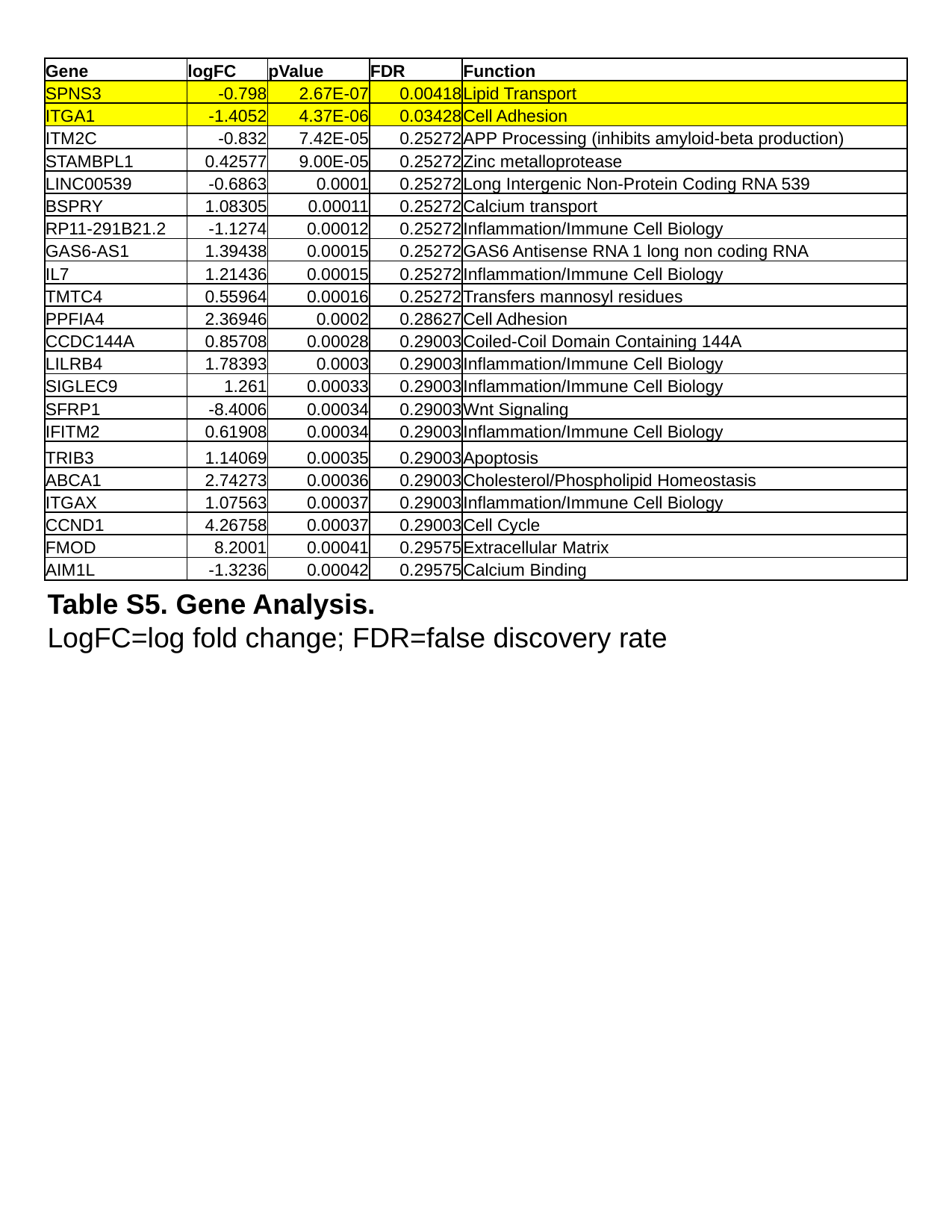

| Gene | logFC | pValue | FDR | Function |
| --- | --- | --- | --- | --- |
| SPNS3 | -0.798 | 2.67E-07 | 0.00418 | Lipid Transport |
| ITGA1 | -1.4052 | 4.37E-06 | 0.03428 | Cell Adhesion |
| ITM2C | -0.832 | 7.42E-05 | 0.25272 | APP Processing (inhibits amyloid-beta production) |
| STAMBPL1 | 0.42577 | 9.00E-05 | 0.25272 | Zinc metalloprotease |
| LINC00539 | -0.6863 | 0.0001 | 0.25272 | Long Intergenic Non-Protein Coding RNA 539 |
| BSPRY | 1.08305 | 0.00011 | 0.25272 | Calcium transport |
| RP11-291B21.2 | -1.1274 | 0.00012 | 0.25272 | Inflammation/Immune Cell Biology |
| GAS6-AS1 | 1.39438 | 0.00015 | 0.25272 | GAS6 Antisense RNA 1 long non coding RNA |
| IL7 | 1.21436 | 0.00015 | 0.25272 | Inflammation/Immune Cell Biology |
| TMTC4 | 0.55964 | 0.00016 | 0.25272 | Transfers mannosyl residues |
| PPFIA4 | 2.36946 | 0.0002 | 0.28627 | Cell Adhesion |
| CCDC144A | 0.85708 | 0.00028 | 0.29003 | Coiled-Coil Domain Containing 144A |
| LILRB4 | 1.78393 | 0.0003 | 0.29003 | Inflammation/Immune Cell Biology |
| SIGLEC9 | 1.261 | 0.00033 | 0.29003 | Inflammation/Immune Cell Biology |
| SFRP1 | -8.4006 | 0.00034 | 0.29003 | Wnt Signaling |
| IFITM2 | 0.61908 | 0.00034 | 0.29003 | Inflammation/Immune Cell Biology |
| TRIB3 | 1.14069 | 0.00035 | 0.29003 | Apoptosis |
| ABCA1 | 2.74273 | 0.00036 | 0.29003 | Cholesterol/Phospholipid Homeostasis |
| ITGAX | 1.07563 | 0.00037 | 0.29003 | Inflammation/Immune Cell Biology |
| CCND1 | 4.26758 | 0.00037 | 0.29003 | Cell Cycle |
| FMOD | 8.2001 | 0.00041 | 0.29575 | Extracellular Matrix |
| AIM1L | -1.3236 | 0.00042 | 0.29575 | Calcium Binding |
Table S5. Gene Analysis.
LogFC=log fold change; FDR=false discovery rate

## Slide 12
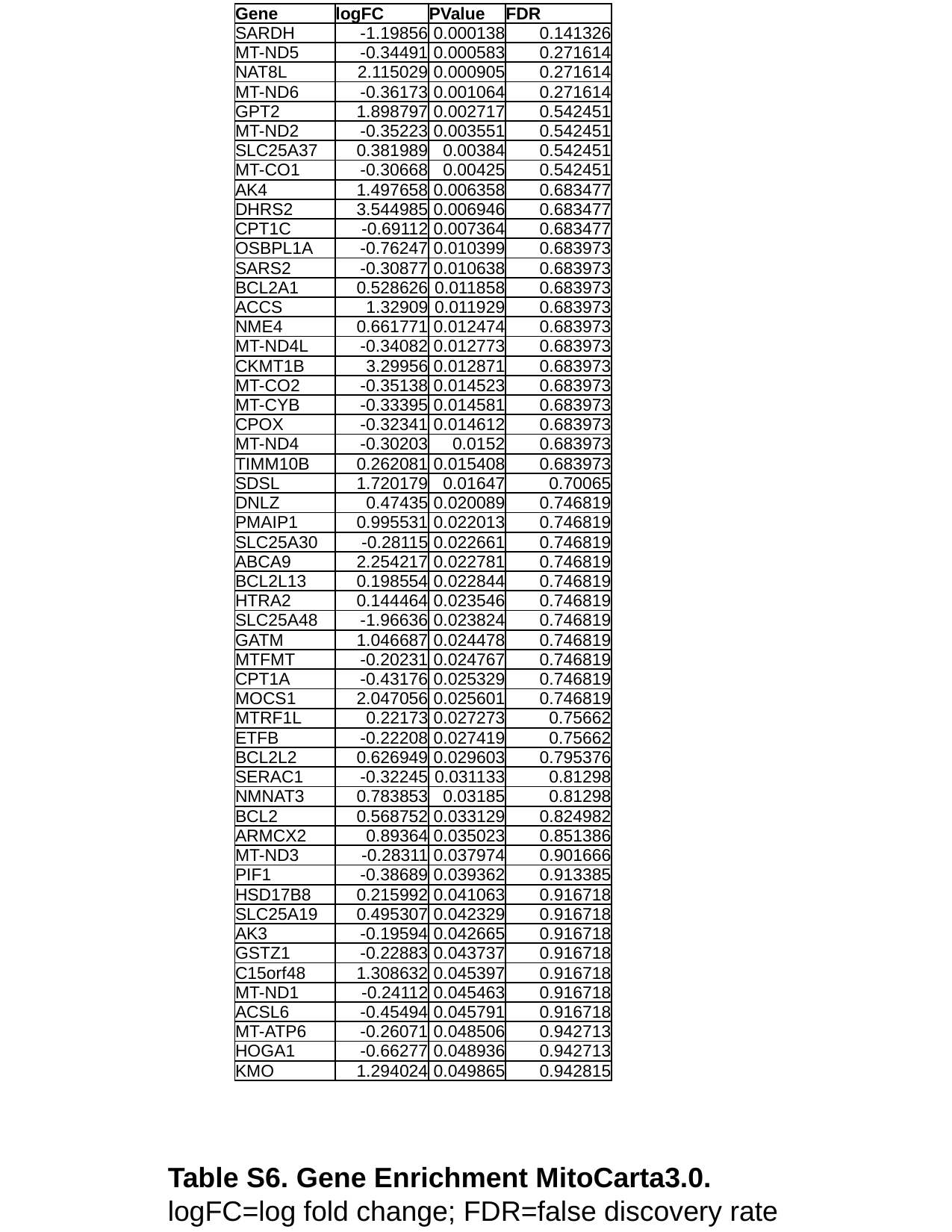

| Gene | logFC | PValue | FDR |
| --- | --- | --- | --- |
| SARDH | -1.19856 | 0.000138 | 0.141326 |
| MT-ND5 | -0.34491 | 0.000583 | 0.271614 |
| NAT8L | 2.115029 | 0.000905 | 0.271614 |
| MT-ND6 | -0.36173 | 0.001064 | 0.271614 |
| GPT2 | 1.898797 | 0.002717 | 0.542451 |
| MT-ND2 | -0.35223 | 0.003551 | 0.542451 |
| SLC25A37 | 0.381989 | 0.00384 | 0.542451 |
| MT-CO1 | -0.30668 | 0.00425 | 0.542451 |
| AK4 | 1.497658 | 0.006358 | 0.683477 |
| DHRS2 | 3.544985 | 0.006946 | 0.683477 |
| CPT1C | -0.69112 | 0.007364 | 0.683477 |
| OSBPL1A | -0.76247 | 0.010399 | 0.683973 |
| SARS2 | -0.30877 | 0.010638 | 0.683973 |
| BCL2A1 | 0.528626 | 0.011858 | 0.683973 |
| ACCS | 1.32909 | 0.011929 | 0.683973 |
| NME4 | 0.661771 | 0.012474 | 0.683973 |
| MT-ND4L | -0.34082 | 0.012773 | 0.683973 |
| CKMT1B | 3.29956 | 0.012871 | 0.683973 |
| MT-CO2 | -0.35138 | 0.014523 | 0.683973 |
| MT-CYB | -0.33395 | 0.014581 | 0.683973 |
| CPOX | -0.32341 | 0.014612 | 0.683973 |
| MT-ND4 | -0.30203 | 0.0152 | 0.683973 |
| TIMM10B | 0.262081 | 0.015408 | 0.683973 |
| SDSL | 1.720179 | 0.01647 | 0.70065 |
| DNLZ | 0.47435 | 0.020089 | 0.746819 |
| PMAIP1 | 0.995531 | 0.022013 | 0.746819 |
| SLC25A30 | -0.28115 | 0.022661 | 0.746819 |
| ABCA9 | 2.254217 | 0.022781 | 0.746819 |
| BCL2L13 | 0.198554 | 0.022844 | 0.746819 |
| HTRA2 | 0.144464 | 0.023546 | 0.746819 |
| SLC25A48 | -1.96636 | 0.023824 | 0.746819 |
| GATM | 1.046687 | 0.024478 | 0.746819 |
| MTFMT | -0.20231 | 0.024767 | 0.746819 |
| CPT1A | -0.43176 | 0.025329 | 0.746819 |
| MOCS1 | 2.047056 | 0.025601 | 0.746819 |
| MTRF1L | 0.22173 | 0.027273 | 0.75662 |
| ETFB | -0.22208 | 0.027419 | 0.75662 |
| BCL2L2 | 0.626949 | 0.029603 | 0.795376 |
| SERAC1 | -0.32245 | 0.031133 | 0.81298 |
| NMNAT3 | 0.783853 | 0.03185 | 0.81298 |
| BCL2 | 0.568752 | 0.033129 | 0.824982 |
| ARMCX2 | 0.89364 | 0.035023 | 0.851386 |
| MT-ND3 | -0.28311 | 0.037974 | 0.901666 |
| PIF1 | -0.38689 | 0.039362 | 0.913385 |
| HSD17B8 | 0.215992 | 0.041063 | 0.916718 |
| SLC25A19 | 0.495307 | 0.042329 | 0.916718 |
| AK3 | -0.19594 | 0.042665 | 0.916718 |
| GSTZ1 | -0.22883 | 0.043737 | 0.916718 |
| C15orf48 | 1.308632 | 0.045397 | 0.916718 |
| MT-ND1 | -0.24112 | 0.045463 | 0.916718 |
| ACSL6 | -0.45494 | 0.045791 | 0.916718 |
| MT-ATP6 | -0.26071 | 0.048506 | 0.942713 |
| HOGA1 | -0.66277 | 0.048936 | 0.942713 |
| KMO | 1.294024 | 0.049865 | 0.942815 |
Table S6. Gene Enrichment MitoCarta3.0.
logFC=log fold change; FDR=false discovery rate

## Slide 13
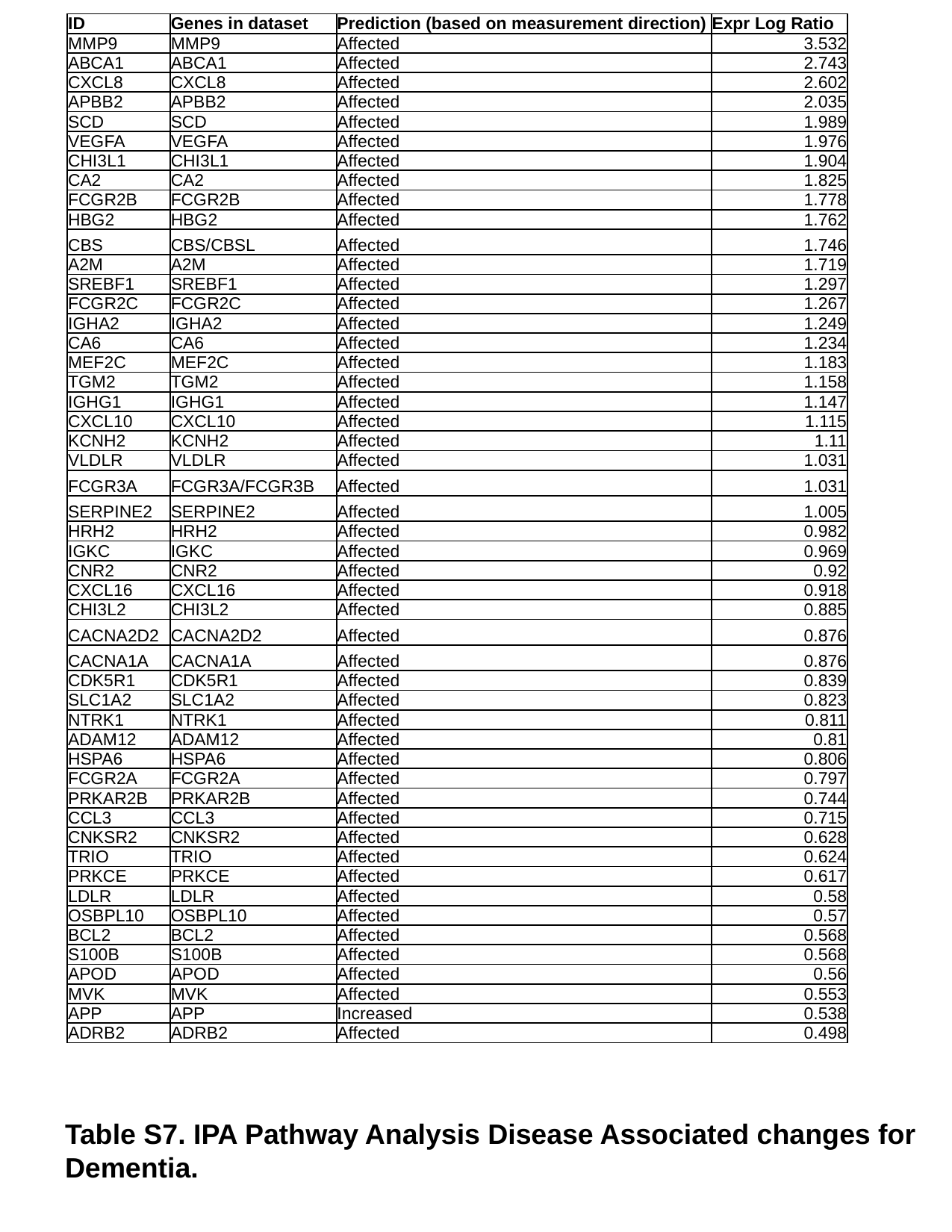

| ID | Genes in dataset | Prediction (based on measurement direction) | Expr Log Ratio |
| --- | --- | --- | --- |
| MMP9 | MMP9 | Affected | 3.532 |
| ABCA1 | ABCA1 | Affected | 2.743 |
| CXCL8 | CXCL8 | Affected | 2.602 |
| APBB2 | APBB2 | Affected | 2.035 |
| SCD | SCD | Affected | 1.989 |
| VEGFA | VEGFA | Affected | 1.976 |
| CHI3L1 | CHI3L1 | Affected | 1.904 |
| CA2 | CA2 | Affected | 1.825 |
| FCGR2B | FCGR2B | Affected | 1.778 |
| HBG2 | HBG2 | Affected | 1.762 |
| CBS | CBS/CBSL | Affected | 1.746 |
| A2M | A2M | Affected | 1.719 |
| SREBF1 | SREBF1 | Affected | 1.297 |
| FCGR2C | FCGR2C | Affected | 1.267 |
| IGHA2 | IGHA2 | Affected | 1.249 |
| CA6 | CA6 | Affected | 1.234 |
| MEF2C | MEF2C | Affected | 1.183 |
| TGM2 | TGM2 | Affected | 1.158 |
| IGHG1 | IGHG1 | Affected | 1.147 |
| CXCL10 | CXCL10 | Affected | 1.115 |
| KCNH2 | KCNH2 | Affected | 1.11 |
| VLDLR | VLDLR | Affected | 1.031 |
| FCGR3A | FCGR3A/FCGR3B | Affected | 1.031 |
| SERPINE2 | SERPINE2 | Affected | 1.005 |
| HRH2 | HRH2 | Affected | 0.982 |
| IGKC | IGKC | Affected | 0.969 |
| CNR2 | CNR2 | Affected | 0.92 |
| CXCL16 | CXCL16 | Affected | 0.918 |
| CHI3L2 | CHI3L2 | Affected | 0.885 |
| CACNA2D2 | CACNA2D2 | Affected | 0.876 |
| CACNA1A | CACNA1A | Affected | 0.876 |
| CDK5R1 | CDK5R1 | Affected | 0.839 |
| SLC1A2 | SLC1A2 | Affected | 0.823 |
| NTRK1 | NTRK1 | Affected | 0.811 |
| ADAM12 | ADAM12 | Affected | 0.81 |
| HSPA6 | HSPA6 | Affected | 0.806 |
| FCGR2A | FCGR2A | Affected | 0.797 |
| PRKAR2B | PRKAR2B | Affected | 0.744 |
| CCL3 | CCL3 | Affected | 0.715 |
| CNKSR2 | CNKSR2 | Affected | 0.628 |
| TRIO | TRIO | Affected | 0.624 |
| PRKCE | PRKCE | Affected | 0.617 |
| LDLR | LDLR | Affected | 0.58 |
| OSBPL10 | OSBPL10 | Affected | 0.57 |
| BCL2 | BCL2 | Affected | 0.568 |
| S100B | S100B | Affected | 0.568 |
| APOD | APOD | Affected | 0.56 |
| MVK | MVK | Affected | 0.553 |
| APP | APP | Increased | 0.538 |
| ADRB2 | ADRB2 | Affected | 0.498 |
Table S7. IPA Pathway Analysis Disease Associated changes for Dementia.

## Slide 14
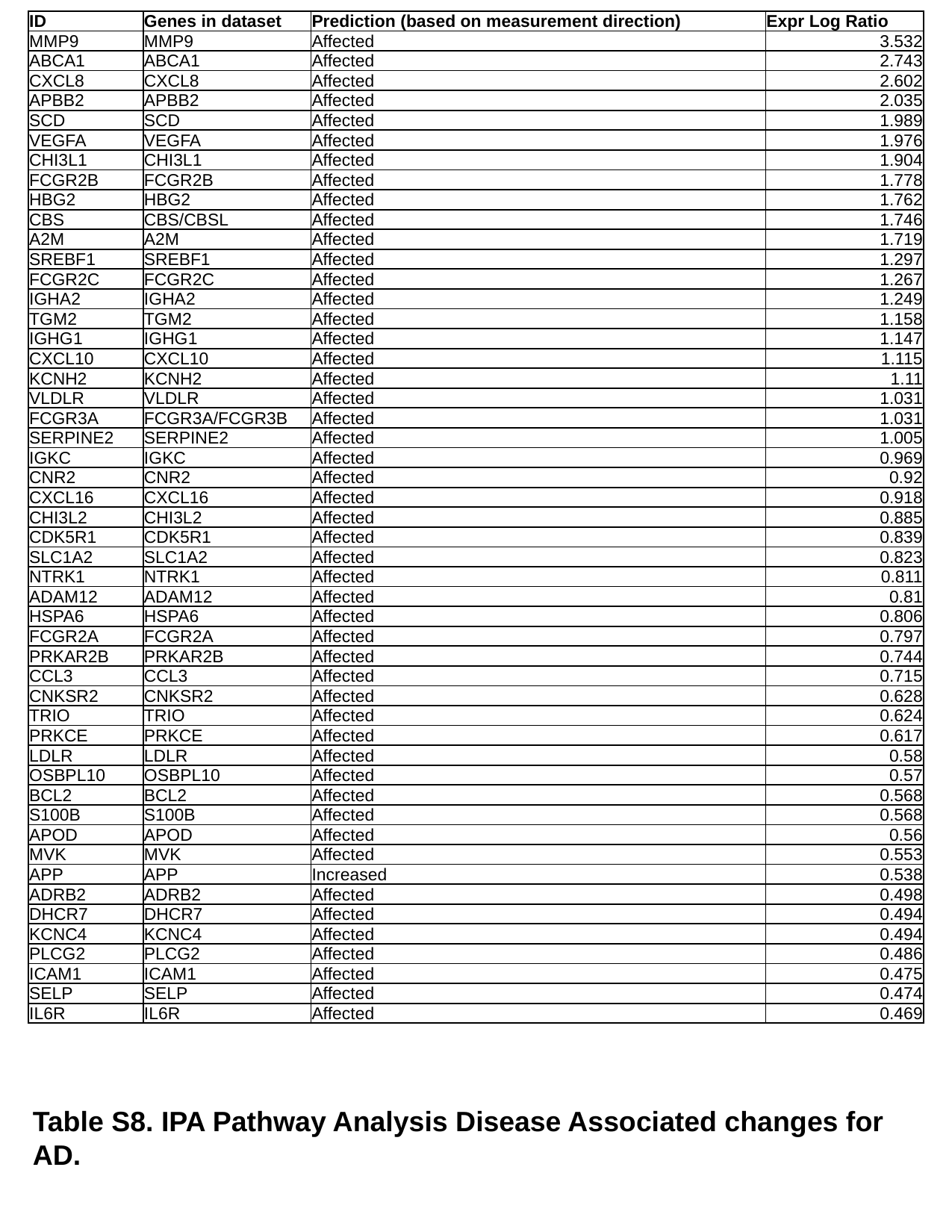

| ID | Genes in dataset | Prediction (based on measurement direction) | Expr Log Ratio |
| --- | --- | --- | --- |
| MMP9 | MMP9 | Affected | 3.532 |
| ABCA1 | ABCA1 | Affected | 2.743 |
| CXCL8 | CXCL8 | Affected | 2.602 |
| APBB2 | APBB2 | Affected | 2.035 |
| SCD | SCD | Affected | 1.989 |
| VEGFA | VEGFA | Affected | 1.976 |
| CHI3L1 | CHI3L1 | Affected | 1.904 |
| FCGR2B | FCGR2B | Affected | 1.778 |
| HBG2 | HBG2 | Affected | 1.762 |
| CBS | CBS/CBSL | Affected | 1.746 |
| A2M | A2M | Affected | 1.719 |
| SREBF1 | SREBF1 | Affected | 1.297 |
| FCGR2C | FCGR2C | Affected | 1.267 |
| IGHA2 | IGHA2 | Affected | 1.249 |
| TGM2 | TGM2 | Affected | 1.158 |
| IGHG1 | IGHG1 | Affected | 1.147 |
| CXCL10 | CXCL10 | Affected | 1.115 |
| KCNH2 | KCNH2 | Affected | 1.11 |
| VLDLR | VLDLR | Affected | 1.031 |
| FCGR3A | FCGR3A/FCGR3B | Affected | 1.031 |
| SERPINE2 | SERPINE2 | Affected | 1.005 |
| IGKC | IGKC | Affected | 0.969 |
| CNR2 | CNR2 | Affected | 0.92 |
| CXCL16 | CXCL16 | Affected | 0.918 |
| CHI3L2 | CHI3L2 | Affected | 0.885 |
| CDK5R1 | CDK5R1 | Affected | 0.839 |
| SLC1A2 | SLC1A2 | Affected | 0.823 |
| NTRK1 | NTRK1 | Affected | 0.811 |
| ADAM12 | ADAM12 | Affected | 0.81 |
| HSPA6 | HSPA6 | Affected | 0.806 |
| FCGR2A | FCGR2A | Affected | 0.797 |
| PRKAR2B | PRKAR2B | Affected | 0.744 |
| CCL3 | CCL3 | Affected | 0.715 |
| CNKSR2 | CNKSR2 | Affected | 0.628 |
| TRIO | TRIO | Affected | 0.624 |
| PRKCE | PRKCE | Affected | 0.617 |
| LDLR | LDLR | Affected | 0.58 |
| OSBPL10 | OSBPL10 | Affected | 0.57 |
| BCL2 | BCL2 | Affected | 0.568 |
| S100B | S100B | Affected | 0.568 |
| APOD | APOD | Affected | 0.56 |
| MVK | MVK | Affected | 0.553 |
| APP | APP | Increased | 0.538 |
| ADRB2 | ADRB2 | Affected | 0.498 |
| DHCR7 | DHCR7 | Affected | 0.494 |
| KCNC4 | KCNC4 | Affected | 0.494 |
| PLCG2 | PLCG2 | Affected | 0.486 |
| ICAM1 | ICAM1 | Affected | 0.475 |
| SELP | SELP | Affected | 0.474 |
| IL6R | IL6R | Affected | 0.469 |
Table S8. IPA Pathway Analysis Disease Associated changes for AD.

## Slide 15
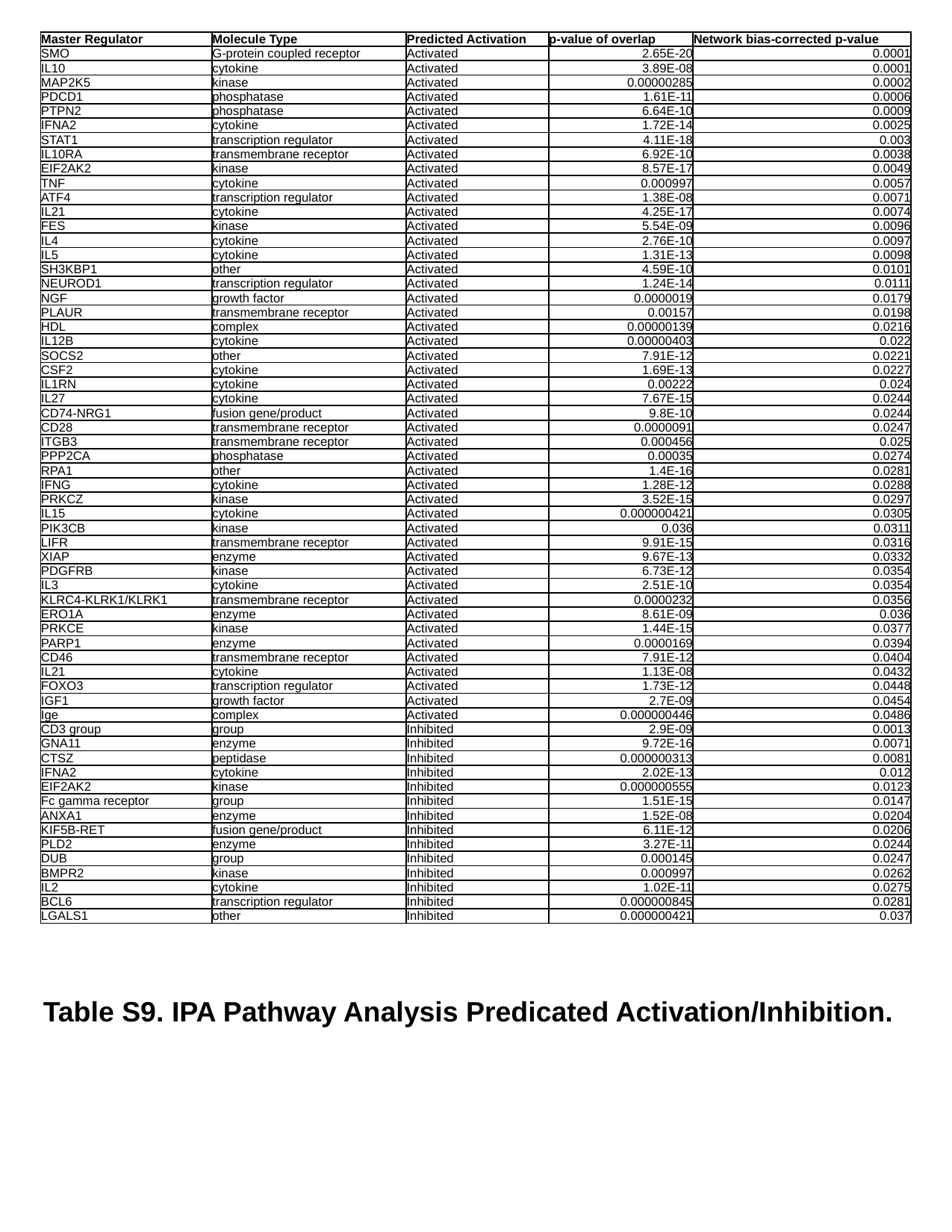

| Master Regulator | Molecule Type | Predicted Activation | p-value of overlap | Network bias-corrected p-value |
| --- | --- | --- | --- | --- |
| SMO | G-protein coupled receptor | Activated | 2.65E-20 | 0.0001 |
| IL10 | cytokine | Activated | 3.89E-08 | 0.0001 |
| MAP2K5 | kinase | Activated | 0.00000285 | 0.0002 |
| PDCD1 | phosphatase | Activated | 1.61E-11 | 0.0006 |
| PTPN2 | phosphatase | Activated | 6.64E-10 | 0.0009 |
| IFNA2 | cytokine | Activated | 1.72E-14 | 0.0025 |
| STAT1 | transcription regulator | Activated | 4.11E-18 | 0.003 |
| IL10RA | transmembrane receptor | Activated | 6.92E-10 | 0.0038 |
| EIF2AK2 | kinase | Activated | 8.57E-17 | 0.0049 |
| TNF | cytokine | Activated | 0.000997 | 0.0057 |
| ATF4 | transcription regulator | Activated | 1.38E-08 | 0.0071 |
| IL21 | cytokine | Activated | 4.25E-17 | 0.0074 |
| FES | kinase | Activated | 5.54E-09 | 0.0096 |
| IL4 | cytokine | Activated | 2.76E-10 | 0.0097 |
| IL5 | cytokine | Activated | 1.31E-13 | 0.0098 |
| SH3KBP1 | other | Activated | 4.59E-10 | 0.0101 |
| NEUROD1 | transcription regulator | Activated | 1.24E-14 | 0.0111 |
| NGF | growth factor | Activated | 0.0000019 | 0.0179 |
| PLAUR | transmembrane receptor | Activated | 0.00157 | 0.0198 |
| HDL | complex | Activated | 0.00000139 | 0.0216 |
| IL12B | cytokine | Activated | 0.00000403 | 0.022 |
| SOCS2 | other | Activated | 7.91E-12 | 0.0221 |
| CSF2 | cytokine | Activated | 1.69E-13 | 0.0227 |
| IL1RN | cytokine | Activated | 0.00222 | 0.024 |
| IL27 | cytokine | Activated | 7.67E-15 | 0.0244 |
| CD74-NRG1 | fusion gene/product | Activated | 9.8E-10 | 0.0244 |
| CD28 | transmembrane receptor | Activated | 0.0000091 | 0.0247 |
| ITGB3 | transmembrane receptor | Activated | 0.000456 | 0.025 |
| PPP2CA | phosphatase | Activated | 0.00035 | 0.0274 |
| RPA1 | other | Activated | 1.4E-16 | 0.0281 |
| IFNG | cytokine | Activated | 1.28E-12 | 0.0288 |
| PRKCZ | kinase | Activated | 3.52E-15 | 0.0297 |
| IL15 | cytokine | Activated | 0.000000421 | 0.0305 |
| PIK3CB | kinase | Activated | 0.036 | 0.0311 |
| LIFR | transmembrane receptor | Activated | 9.91E-15 | 0.0316 |
| XIAP | enzyme | Activated | 9.67E-13 | 0.0332 |
| PDGFRB | kinase | Activated | 6.73E-12 | 0.0354 |
| IL3 | cytokine | Activated | 2.51E-10 | 0.0354 |
| KLRC4-KLRK1/KLRK1 | transmembrane receptor | Activated | 0.0000232 | 0.0356 |
| ERO1A | enzyme | Activated | 8.61E-09 | 0.036 |
| PRKCE | kinase | Activated | 1.44E-15 | 0.0377 |
| PARP1 | enzyme | Activated | 0.0000169 | 0.0394 |
| CD46 | transmembrane receptor | Activated | 7.91E-12 | 0.0404 |
| IL21 | cytokine | Activated | 1.13E-08 | 0.0432 |
| FOXO3 | transcription regulator | Activated | 1.73E-12 | 0.0448 |
| IGF1 | growth factor | Activated | 2.7E-09 | 0.0454 |
| Ige | complex | Activated | 0.000000446 | 0.0486 |
| CD3 group | group | Inhibited | 2.9E-09 | 0.0013 |
| GNA11 | enzyme | Inhibited | 9.72E-16 | 0.0071 |
| CTSZ | peptidase | Inhibited | 0.000000313 | 0.0081 |
| IFNA2 | cytokine | Inhibited | 2.02E-13 | 0.012 |
| EIF2AK2 | kinase | Inhibited | 0.000000555 | 0.0123 |
| Fc gamma receptor | group | Inhibited | 1.51E-15 | 0.0147 |
| ANXA1 | enzyme | Inhibited | 1.52E-08 | 0.0204 |
| KIF5B-RET | fusion gene/product | Inhibited | 6.11E-12 | 0.0206 |
| PLD2 | enzyme | Inhibited | 3.27E-11 | 0.0244 |
| DUB | group | Inhibited | 0.000145 | 0.0247 |
| BMPR2 | kinase | Inhibited | 0.000997 | 0.0262 |
| IL2 | cytokine | Inhibited | 1.02E-11 | 0.0275 |
| BCL6 | transcription regulator | Inhibited | 0.000000845 | 0.0281 |
| LGALS1 | other | Inhibited | 0.000000421 | 0.037 |
Table S9. IPA Pathway Analysis Predicated Activation/Inhibition.

## Slide 16
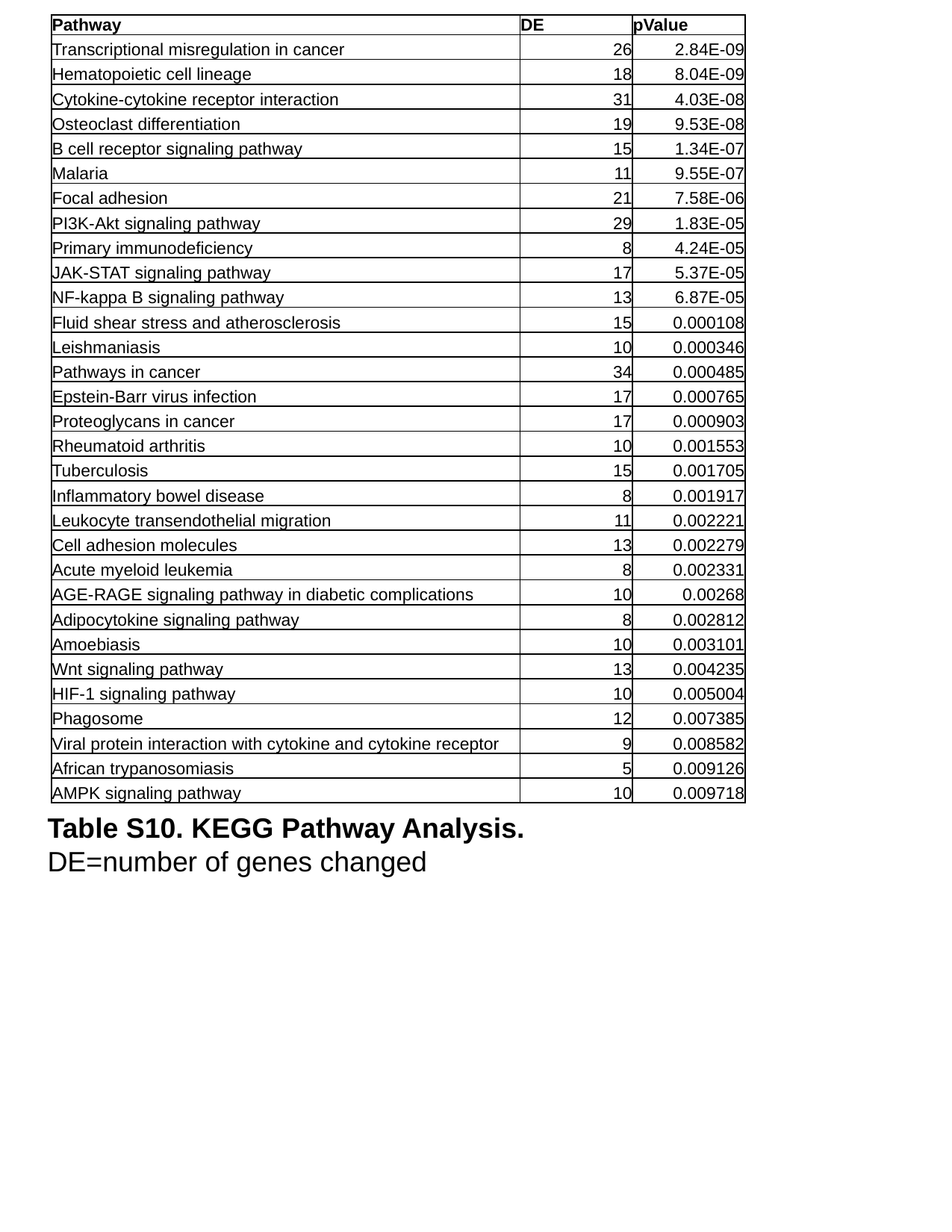

| Pathway | DE | pValue |
| --- | --- | --- |
| Transcriptional misregulation in cancer | 26 | 2.84E-09 |
| Hematopoietic cell lineage | 18 | 8.04E-09 |
| Cytokine-cytokine receptor interaction | 31 | 4.03E-08 |
| Osteoclast differentiation | 19 | 9.53E-08 |
| B cell receptor signaling pathway | 15 | 1.34E-07 |
| Malaria | 11 | 9.55E-07 |
| Focal adhesion | 21 | 7.58E-06 |
| PI3K-Akt signaling pathway | 29 | 1.83E-05 |
| Primary immunodeficiency | 8 | 4.24E-05 |
| JAK-STAT signaling pathway | 17 | 5.37E-05 |
| NF-kappa B signaling pathway | 13 | 6.87E-05 |
| Fluid shear stress and atherosclerosis | 15 | 0.000108 |
| Leishmaniasis | 10 | 0.000346 |
| Pathways in cancer | 34 | 0.000485 |
| Epstein-Barr virus infection | 17 | 0.000765 |
| Proteoglycans in cancer | 17 | 0.000903 |
| Rheumatoid arthritis | 10 | 0.001553 |
| Tuberculosis | 15 | 0.001705 |
| Inflammatory bowel disease | 8 | 0.001917 |
| Leukocyte transendothelial migration | 11 | 0.002221 |
| Cell adhesion molecules | 13 | 0.002279 |
| Acute myeloid leukemia | 8 | 0.002331 |
| AGE-RAGE signaling pathway in diabetic complications | 10 | 0.00268 |
| Adipocytokine signaling pathway | 8 | 0.002812 |
| Amoebiasis | 10 | 0.003101 |
| Wnt signaling pathway | 13 | 0.004235 |
| HIF-1 signaling pathway | 10 | 0.005004 |
| Phagosome | 12 | 0.007385 |
| Viral protein interaction with cytokine and cytokine receptor | 9 | 0.008582 |
| African trypanosomiasis | 5 | 0.009126 |
| AMPK signaling pathway | 10 | 0.009718 |
Table S10. KEGG Pathway Analysis.
DE=number of genes changed
